# Osteoarthritis Bone Marrow MSCs Retain Regenerative Competence and Chemokine Responsiveness for Drug‐Based In Situ Tissue Engineering

**DOI:** 10.1155/sci/3757831

**Published:** 2025-12-30

**Authors:** Julia Sonnleitner, Katja Gulich, Axel Pruss, Carsten Perka, Angelika Gursche, Daniel Kendoff, Michael Sittinger, Shabnam Hemmati-Sadeghi, Tilo Dehne

**Affiliations:** ^1^ Tissue Engineering Laboratory, Department for Rheumatology and Clinical Immunology, Charité–Universitätsmedizin Berlin, Charitéplatz 1, Berlin, 10117, Germany, charite.de; ^2^ Institute of Transfusion Medicine, University Tissue Bank, Charité–Universitätsmedizin Berlin, Berlin, 10117, Germany, charite.de; ^3^ Center for Musculoskeletal Surgery, Charité–Universitätsmedizin Berlin, Berlin, 10117, Germany, charite.de; ^4^ Department of Orthopaedic Surgery, Helios Klinikum Berlin-Buch, Berlin, 13125, Germany, helios-kliniken.de

## Abstract

**Background:**

Mesenchymal stromal cells (MSCs) support tissue repair in osteoarthritis (OA), with migration to damaged tissue being a key strategy in in situ tissue engineering. Their regenerative potential depends on factors such as differentiation, level of senescence, and responsiveness to signaling molecules. However, previous findings on these properties of OA MSCs remain inconclusive. This study integrates multiple aspects and tests feasibility using a well‐characterized chemoattractant.

**Methods and Results:**

MSCs from non‐OA donor (ND) and OA donor were characterized for their trilineage differentiation potential as well as for their senescence level by (immune‐) histochemistry, RT‐qPCR, microarray analysis, and a bead‐based immunoassay for cell culture supernatants. No difference in differentiation and senescence level was observed, the latter being indicated by a similar activity of *β*‐Galactosidase (*β*‐gal), gene expression profiles of cyclin‐dependent kinase (CDKN) inhibitor 2A (CDKN2A), CDKN inhibitor 1A (CDKN1A), sirtuin 1 (SIRT1), and matrix metallopeptidase 1 (MMP1), as well as secreted cytokines. Chemokine receptors in OA MSCs were detected using immunohistochemistry and RT‐qPCR. Expression of CCR1‐CCR7, CCR9, and CXCR1‐CXCR6 in OA MSCs was confirmed on gene and protein levels. Both OA and ND MSCs migrated toward 1000 nM CCL25, as evaluated via a Boyden chamber assay. Subsequent genome‐wide microarray analysis of OA MSCs after treatment with 1000 nM CCL25 corroborated its influence on migration, proliferation, apoptosis, and differentiation as defined by Gene Ontology terms (GO terms). Kyoto Encyclopedia of Genes and Genomes (KEGG) pathway analysis confirmed this broad impact and emphasized the role of cytokine–cytokine–receptor interaction and metabolic pathways.

**Conclusion:**

Our data indicate that OA MSCs retain their differentiation potential and do not exhibit an increased senescent phenotype. Their chemokine receptor profile is conducive of migration, and both OA and ND MSCs respond to CCL25, highlighting the potential of OA MSCs for directed in situ repair.

## 1. Introduction

Osteoarthritis (OA) is a chronic, multifactorial disease of the joint, which involves synovial inflammation, bone remodeling, and cartilage destruction [1]. It poses a serious burden of global disease, with an incidence rate that has increased by 9.3% between 1990 and 2017 [2]. The prevalence of knee OA in people older than 40 years is estimated to be around 23% and to be further increasing with an aging population [3]. Individual and societal costs are high, especially considering comorbidities and productivity loss [4, 5].

The typical therapeutic strategy is merely symptomatic and focuses primarily on exercise, dietary measurements, and pain management [6], eventually followed by operative joint replacement [7]. While alternative approaches like autologous chondrocyte transplantation, intra‐articular injection of mesenchymal stromal cells (MSCs), and microfracturing have yielded partly satisfactory results, their application requires two‐stage procedures, ex vivo proliferation, or surgical interventions and is thus accompanied by considerable financial and logistical costs.

In situ tissue engineering represents a strategy that takes advantage of inherent physiological repair mechanisms and seeks to enhance them [8]. Cell‐free scaffolds or hydrogels containing chemoattractants or growth factors, which are released in a spatio‐temporal manner, optimize the homing of endogenous cells and contribute to tissue regeneration [9–11]. A strong focus has been placed on MSCs as a source for endogenous regeneration, as they have been shown to contribute to tissue recovery in OA and other pathologies like myocardial infarction and injured liver or pancreas tissue [12–14]. MSCs reside in numerous niches, can migrate toward chemokines and cytokines, and differentiate into adipocytes, osteoblasts, and chondrocytes following the corresponding stimulus [8]. Additionally, MSCs can halt and reverse the degeneration of surrounding tissue by secreting pro‐regenerative factors [15, 16] and modulating the immune system [17]. Several growth factors and chemokines have been described to recruit MSCs [18] and contribute to articular cartilage regeneration, for example interleukin (IL)‐8 (IL‐8; CXCL8), macrophage inflammatory protein‐3*α* (MIP3*α*; CCL20), and stromal derived factor‐1 (SDF1; CXCL12) [19–21], emphasizing the importance of chemokine receptors as mediators of this effect.

In 1997, Vicari et al. identified a new chemokine in the thymus, hence named thymus‐expressed chemokine (TECK) [22], now also known as CCL25, which binds to CCR9 as its only known ligand [23]. The CCL25/CCR9 interaction involves a broad range of biological processes, from T‐cell development and chemotaxis of macrophages and dendritic cells [22] to inflammation‐related disease and cancer [24, 25]. Our group has previously demonstrated the chemotactic effect CCL25 exerts on MSCs from healthy donors at a concentration of around 1000 nM [26–28]. In addition to its homing ability, in vivo evidence suggests a pro‐regenerative effect on cartilage [29], making CCL25 a novel candidate for in situ tissue engineering, which not only recruits MSCs but potentially contributes to cartilage regeneration. While sufficient migration is one requirement for in situ tissue engineering, another is the preserved differentiation potential and pro‐regenerative function of MSCs from OA patients. OA is a disease affecting the whole joint. Changes in subchondral and trabecular bone are a well‐described hallmark in OA, featuring bone marrow lesions directly associated with cartilage damage [30], altered cellular composition, and increased TGF‐*β* levels [31], possibly affecting the properties of MSCs. Conflicting evidence exists on how or if OA alters the aforementioned properties of MSCs and thus potentially impairs the outcome of in situ tissue engineering approaches. Specifically regarding MSCs from bone marrow, some studies indicated reduced proliferation and differentiation of MSCs from OA donors (OA MSCs) compared to those from non‐OA donors (ND MSCs) [32, 33], while others did not find such a difference [34–36]. Moreover, a potential senescent phenotype of OA MSCs could impose a major limitation on their regenerative potential. Senescence is defined as a cellular state of irreversible cell cycle arrest, characterized by features including increased activity of *β*‐Galactosidase (*β*‐gal) and altered gene expression and mitochondrial dysfunction. It plays a role in a myriad of biological processes, like embryogenesis, wound healing, and cancer development. Senescent cells can also act as a driver for aging‐related disease [37, 38], not only due to an impaired function of the cell itself but also through paracrine effects on the surrounding tissue via the senescence‐associated secretory phenotype (SASP) [39]. As for OA, the presence of senescent MSCs in cartilage has been previously reported [40, 41], and it was demonstrated in vitro and in an animal model that senescent MSCs contribute to OA progression [42]. At the same time, the attenuation of senescence had a beneficial impact on the progression of OA [43, 44]. So far, the connection between OA and its impact on MSCs that are not within close proximity to cartilage remains tenuous. Mareddy et al. [45] examined both fast and slow‐growing MSCs in bone marrow from OA patients, with the former showing no restriction, while the latter exhibited impaired differentiation and signs of senescence. Another study did not find markers for senescence comparing OA to ND MSCs [33].

As OA and its effects—on both individuals and society—become more prevalent, the quest for new therapeutic options has become increasingly important. The field of in situ tissue engineering offers novel and scalable methods that leverage the body’s own regenerative functions. The following study examined and corroborated essential qualifications of bone marrow MSCs from OA donors for this approach by analyzing their differentiation capacity and level of senescence and characterizing their chemokine receptor profile. After confirming the migration of OA MSCs toward CCL25, we explored its impact on genome‐wide gene expression to pave the way for another chemokine that might be utilized therapeutically as an in situ chemoattractant. Therefore, this study investigates whether OA bone marrow‐derived MSCs preserve their intrinsic regenerative competence and maintain chemokine responsiveness that together constitute key prerequisites for drug‐based in situ tissue engineering strategies aiming to mobilize and direct endogenous MSCs for joint repair.

## 2. Methods

### 2.1. Isolation and Cultivation of MSCs

This procedure was approved by the ethical committee of the Charité – Universitätsmedizin Berlin (EA2/068/14) and by the patient who gave informed consent. OA MSCs were isolated as previously described [46] from cancellous bone of femoral heads from patients undergoing hip replacement due to OA. ND MSCs were obtained from human bone marrow that was harvested by aspiration of the iliac crest from patients for diagnostic reasons or due to fractures. MSCs from 11 different ND (four of them female, mean age 64 years, SD ± 18 years) and 13 different OA donors (nine of them female, mean age 65 years, SD ± 9 years) were used for subsequent experiments. Primary cells were cultured at 37°C and 5% CO_2_; medium was changed every 2–3 days. Cell culture medium Dulbecco’s Modified Eagle Medium (DMEM 1 g/L glucose; Biochrom, Schaffhausen, Switzerland) was supplemented with 10% fetal bovine serum (FBS; HyClone, Cramlington, UK), 20 mM HEPES (Biochrom), 2 mM L‐glutamine (Biochrom), 1% penicillin (100 units/mL)/streptomycin (100 μg/mL) (Sigma–Aldrich, St. Louis, MO, USA) and 2 ng/mL human basic‐fibroblast growth factor (bFGF; Pepro Tech, London, UK)—subsequently referred to as complete DMEM.

Monolayer MSCs were detached with 0.05% trypsin/EDTA (Biochrom) at 90% confluency, reseeded at 5 × 10^3^ cells/cm^2^, and expanded to Passage 2 or 3 for experimental use. All experiments were conducted with three individual donors. To quantify the proliferation potential, MSCs were cultured up to Passage 4, counted before each Passage, and the specific growth rate μ was calculated using the following formula:

μ=lnNt/N0t.

### 2.2. Analysis of Trilineage Differentiation Potential of MSCs

MSCs at Passage 3 were characterized by their ability to differentiate into adipogenic, chondrogenic, and osteogenic lineages. For adipogenic differentiation, MSCs were detached, plated at a density of 10^4^ cells/cm^2^, and cultivated up to postconfluence. Subsequently, they were treated with induction medium composed of high glucose DMEM (4.5 g/L glucose, Biochrom) containing 10% FBS (HyClone), 1% penicillin/streptomycin (Sigma–Aldrich), 20 mM HEPES (Biochrom), 10 µg/mL insulin Actraphane (Novo Nordisk, Mainz, Germany), 1 µM dexamethasone (Sigma–Aldrich), 200 µM indomethacin (Sigma–Aldrich), and 50 µM 3‐isobutyl‐1‐methylxanthine (IBMX; Sigma–Aldrich) for 3 days followed by 2 days of cultivation in maintenance medium consisting of high glucose DMEM (4.5 g/L glucose; Biochrom) supplemented with 10% FBS (HyClone), 1% penicillin/streptomycin (Sigma–Aldrich), 20 mM HEPES (Biochrom) and 10 µg/mL insulin Actraphane (Novo Nordisk). One cycle consisting of 3 days of induction followed by 2 days of maintenance was repeated three times. MSCs cultivated only in maintenance medium served as negative control. Cellular lipid vacuoles of adipocytes were identified by Oil Red O staining (Sigma–Aldrich) on days 0 and 15.

Chondrogenic differentiation was performed as previously described [47]. Briefly, 2.5 × 10^5^ MSCs were harvested, centrifuged at 150 *g* for 5 min to form high‐density pellets and treated for 28 days with induction medium consisting of high glucose DMEM (4.5 g/L; Biochrom) supplemented with 1% penicillin/streptomycin (Sigma–Aldrich), 20 mM HEPES (Biochrom), 0.1 µM dexamethasone (Sigma–Aldrich), 1 mM sodium pyruvate (Sigma–Aldrich), 0.17 mM L‐ascorbic acid‐2‐phosphate (Sigma–Aldrich), 0.35 mM proline (Sigma–Aldrich), 1% v/v insulin‐transferrin‐selenium + 1 (ITS + 1; Sigma–Aldrich), and 10 ng/mL of transforming growth factor *β* 3 (TGF‐beta 3; R&D Systems, Minneapolis, MN, USA) as chondrogenic inductor. Medium was changed by 90% every 2 or 3 days. Control pellets were cultured without TGF‐beta 3. Sections (8 µm) of high‐density pellets were stained with Alcian blue 8GX (Roth, Karlsruhe, Germany) to confirm chondrogenesis by identifying acid mucosubstances. Nuclei were counterstained with Nuclear fast red aluminum sulfate solution (Roth). The BERN score was applied to compare chondrogenic matrix formation of ND and OA MSCs [48]. For this purpose, sections were stained with Safranin O (Sigma–Aldrich), staining nonmineralized cartilage and proteoglycans red, while Fast green (Sigma–Aldrich) was used to detect cytoplasm and nuclear membranes. BERN score included the assessment of Safranin O staining intensity, cell distance, and morphology. The EnVision horseradish peroxidase (HRP) immunohistochemistry system (rabbit; DAKO, Santa Clara, CA, USA) was used to detect type II collagen with a rabbit primary antibody against human type II collagen alpha 1 chain (Acris, Hiddenhausen, Germany) and a secondary anti‐rabbit antibody conjugated with HRP (DAKO). Rabbit immunoglobulin fraction (DAKO) served as isotype control. Nuclei were counterstained by hamatoxylin (Merck, Darmstadt, Germany).

For osteogenic differentiation, MSCs were detached, plated at a density of 1 × 10^4^ cells/cm^2^, and cultivated up to 90% confluence. Osteogenesis was induced by the addition of an induction medium consisting of complete DMEM (1 g/L glucose; Biochrom) lacking human FGF basic, supplemented with 0.1 µM dexamethasone, 0.05 mM L‐ascorbic acid‐2‐phosphate, and 10 mM *β*‐glycerophosphate (all Sigma–Aldrich) for 28 days. Medium was changed every 2–3 days. For analysis of osteogenesis, the activity of osteoblast‐typical enzyme alkaline phosphatase (AP) was determined using NBT/BCIP tablets (Roche Diagnostics, Mannheim, Germany), and forming of extracellular mineralized matrix was analyzed by von Kossa staining on days 0 and 28. Differentiation was further evaluated by conducting semiquantitative scoring for Oil Red O, AP, and von Kossa staining. Hereby, + stands for strong, (+) for weak, and − for no staining compared to the negative control.

### 2.3. Analysis of Surface Markers Using Flow Cytometry

Detached MSCs (Passage 3) were washed with PBS/0.5% bovine serum albumin (BSA, Fraction V, Sigma–Aldrich). For analysis of MSCs typical antigen pattern via flow cytometry, 2.5 × 10^5^ cells were incubated separately with R‐phycoerythrin (PE)‐labeled anti‐human CD14 (1:10), CD34 (1:50), CD73 (1:50), CD166 (1:10) and fluorescein isothiocyanate (FITC)‐labeled anti‐human CD44 (1:20), CD45 (1:20), CD90 (1:20), CD105 (1:20) at 4°C for 15 min in the dark (all from BD Pharmingen, except CD105 from Acris). Incubation with respective antibodies was performed at 4°C for 45 min in the dark. Unbound antibody was removed by a final washing step with PBS/0.5% BSA. Immediately before measurement, propidium iodide (0.1 mg/mL) (BD Pharmingen) was added to exclude dead cells from further analysis. Fluorescence intensity was recorded for 1.5 × 10^4^ events per sample. Expression analysis was performed by CellQuestPro 6.0 software (BD Pharmingen).

### 2.4. Immunocytochemical Staining of Chemokine Receptors

Immunocytochemical staining of chemokine receptors of monolayer OA MSCs was performed with the EnVision System (mouse, DAKO). OA MSCs from three donors (Passage 3) were seeded in 8‐well chamber slides (Thermo Fisher Scientific, Nunc, Waltham, MA, USA) and cultured for 24 h. After fixation with methanol/acetone (1:1 v/v), cells were incubated for 30 min at 37°C with unconjugated primary antibodies or control mouse‐IgG1 antibody fraction. Primary antibodies CCR1‐CCR3, CCR5‐7, CCR9, CXCR1‐6 were purchased from R&D‐Systems, CCR4 from BD Pharmingen, CCR8, CCR10, and CX_3_CR from DPC Biermann (Bad Nauheim, Germany). For detection, HRP‐labeled secondary antibodies (DAKO) were applied according to the manufacturer’s protocol. Cells were counterstained with hematoxylin.

### 2.5. Chemotaxis Assays

The recruiting effect of CCL25 on ND and OA MSCs was assessed by Boyden chamber migration assay. This assay also screened for an appropriate chemokine concentration for subsequent CCL25‐mediated gene expression analysis.

The chemotactic response of cells was tested using 8 μm pore‐size polycarbonate membranes in 96‐multiwell ChemoTx plates (Neuroprobe, Gaithersburg, MD, USA). A volume of 37.5 µL chemokine (all purchased from PeproTech) diluted in deprivation medium (DMEM containing 0.1% FBS) was added in triplicates at 1, 10, 100, 1000 nM to wells of the lower compartment. The membrane was fixated on top of the lower wells. MSCs (Passage 3) were detached, washed twice in deprivation medium, and seeded at 3 × 10^4^ cells in 40 µL in wells of the upper compartment. Deprivation medium served as negative control. Cells were allowed to migrate for 20 h at 37°C and 5% CO_2_. After incubation, nonadherent cells were removed, and cells adhering to the lower side of the membrane were fixed in ice‐cold methanol/acetone (1:1 v/v), stained with Hemacolor Staining Kit (Merck), and enumerated microscopically by counting four representative fields. The chemotactic index was determined by normalizing the number of migrated cells per well to the number of migrated cells in the negative control.

### 2.6. Proliferation and Apoptosis in Response to CCL25

About 6 × 10^5^ OA MSCs were seeded in 25 cm^2^ flasks and treated with 1000 nM CCL25 (PeproTech) in deprivation medium or in deprivation medium only at 37°C and 5% CO_2_. After 20 h, cells were washed with PBS and detached with 0.05% trypsin/EDTA. For the analysis of proliferation, 0.125 × 10^5^ cells were reseeded in 25 cm^2^ flasks, cultured until reaching confluency, and subsequently trypsinized, counted, and reseeded at the same density. Population doubling (PD) was calculated to assess the proliferative capacity of OA MSCs. To determine apoptosis of OA MSCs after 20 h treatment with 1000 nM CCL25, Annexin V and PI were stained according to the manufacturer’s instructions (Thermo Fisher Scientific) and analyzed using flow cytometry (FACS BD Canto II). The results were evaluated using FlowJo v10.8 Software (BD Life Sciences). Cells were treated with CCL25 as described above. After trypsinization, they were incubated at 37°C and 5% CO_2_ in suspension in cell culture medium for 30 min and subsequently centrifuged at 390xg for 5 min, washed in cold PBS, centrifuged again, and resuspended in 1× annexin‐binding buffer (all Thermo Fisher Scientific) at a concentration of 1 × 10^6^ cells/mL. About 5 μL of FITC Annexin V and 1 μL of PI (0.1 mg/mL) were added to each 100 μL cell suspension and incubated for 15 min before analysis. Cells incubated at 55°C and 400 rpm for 30 min on a ThermoMixer C (Eppendorf) served as positive control. Gating strategies are shown in Supporting Information 1.

### 2.7. Gene Array—Gene Expression Profiling of CCL25‐Stimulated OA MSCs and for Comparison of ND and OA MSCs

For RNA isolation and subsequent gene expression profiling of CCL25‐stimulated OA MSCs, cells were detached at the end of Passage 3 and 6 × 10^5^ cells were seeded in 25 cm^2^ flasks and stimulated with 1000 nM CCL25 deprivation medium at 37°C and 5% CO_2_. Controls were incubated in deprivation medium only. After 20 h, cells were lysed with TriReagent (Sigma–Aldrich). To compare ND and OA MSCs, cells in monolayer were also lysed with TriReagent. All samples were subsequently processed as described. For phase separation, 1‐bromo‐3‐chloropropane (Sigma–Aldrich) was added to the samples (133 µL/mL TRI‐reagent). After centrifugation, the upper phase was transferred and mixed with 70% ethanol (1:1 v/v). Total cellular RNA isolation was performed with the RNeasy Mini Kit (Qiagen) according to the manufacturer’s protocol. Prior to further processing of total RNA for microarray analysis, RNA integrity was assessed using the Agilent RNA 6000 nano‐Kit according to the manufacturer’s protocol with the 2100 Bioanalyzer (Agilent Technologies).

Total RNA of the three donors per experimental group was used for genome‐wide microarray analysis with the Affymetrix HG‐U133 plus 2.0 array (Affymetrix) according to the manufacturer’s recommendations. About 200 ng of total RNA was used to synthesize biotin‐labeled cRNA. Next, 15 μg of fragmented cRNA was hybridized to gene chips for 16 h at 45°C. Gene chips were washed and stained as recommended. Chips were scanned with the GeneArray scanner controlled by Affymetrix GCOS 1.4 software (Affymetrix). Raw gene expression data were processed and normalized with the GCOS 1.4 software.

An unsupervised approach for CCL25‐treated samples was conducted. Three pairwise comparisons were performed comparing the three CCL25‐treated samples with the three controls. A significance level (change call) is calculated based on match‐mismatch ratios. Samples were considered significant when nine out of nine comparisons showed a significant change call or when *p*  < 0.01 when applying Student’s *t*‐test on signal values. Significantly differentially expressed genes (DEGs) with a fold change > 2 or < −2 were used for further analysis, yielding a total of 777 DEGs.

Analysis of Gene Ontology terms (GO terms) for biological processes and Kyoto Encyclopedia of Genes and Genomes (KEGG) pathways was performed to explore functional implications of DEGs using Enrichr [49, 50]. An adjusted *p*‐value of  < 0.05 was considered significant for both enriched GO terms and enriched KEGG pathways. For GO terms, all DEGs were used as input data. For KEGG pathways, up‐ and downregulated DEGs were examined separately, based on the finding of Hong et al. [51] showing that split analyses lead to more significantly enriched and disease‐relevant pathways.

A hypothesis‐driven approach was chosen for the comparison of ND with OA MSCs. Our aim was to cover a comprehensive spectrum of genes potentially involved in senescence. To achieve this, we consulted two reviews addressing widely acknowledged senescence markers [37, 39], one research paper specifically focusing on senescence in MSCs [52], and two whole transcriptome analyses based on different senescence‐inducing stimuli as well as cell types [53, 54]. From these resources, we compiled a list of senescence‐associated genes. The whole gene expression dataset was then screened for these 187 genes (Supporting Information 2). Significant detection of more than 50% of samples, as calculated by the match–mismatch ratio used in Affymetrix gene arrays, served as the requirement for further analysis, resulting in 157 genes. A significance threshold was determined at *p*  < 0.05 when applying Student’s *t*‐test on signal values.

Microarray data have been deposited in the National Center for Biotechnology Information Gene Expression Omnibus (GEO) and are accessible through GEO series accession numbers GSE267908 regarding the data of OA and ND MSCs and through GSE267906 regarding the analysis of the effect of CCL25 on OA MSCs.

### 2.8. qPCR—Gene Expression Profiling of Chemokine Receptors and Senescence Markers

#### 2.8.1. RNA‐Isolation and cDNA‐Synthesis

MSCs at the end of Passage 2 and MSCs 48 h after seeding cells in Passage 3 were washed with PBS and lysed with 1 mL TRIReagent (Sigma–Aldrich) for analysis of chemokine receptors and senescence markers, respectively. Samples were stored at −80°C for further experimental use.

For samples thawed at room temperature, 133 μL 1‐bromo‐3‐chloropropane (Sigma–Aldrich) was added per milliliter TriReagent LS (Sigma–Aldrich), shaken per hand for 20 s and then for 30 min on an Intelli mixer (NeoLab, Heidelberg, Germany) at 1350 rpm. Samples were centrifuged for 60 min at 17,000 *g* at 4°C, and the upper transparent phase containing RNA was collected. RNA was isolated using RNeasy Mini Spin Columns (Qiagen) according to the manufacturer’s protocol. RNA was quantified via NanoDrop (NanoDrop Technologies, Wilmington, DE, USA), and ratios of absorbance at 260/280 and 260/230 were assessed. About 1 µg of RNA was transcribed to cDNA using the iScript cDNA Synthesis Kit (Bio‐Rad Laboratories, Hercules, CA, USA) according to the manufacturer’s instructions. Briefly, RNA samples were incubated for 5 min at 65°C in the iCycler iQ (Bio‐Rad Laboratories) before adding 5× iScript Reaction Mix and iScript Reverse Transcriptase, followed by the subsequent program in the iCycler iQ (Bio Rad‐Laboratories): 5 min at 25°C, 30 min at 42°C, 5 min at 85°C, and then held at 4°C until stored at −20°C before further experimental use.

#### 2.8.2. Real‐Time qPCR

For receptor oligonucleotide sequences used, see Supporting Information 3. For the analysis of chemokine receptors, real‐time qPCR was performed using the SYBR Green PCR Core Kit (Applied Biosystems, Karlsruhe, Germany) and i‐Cycler system (Bio‐Rad Laboratories) according to the manufacturer’s protocol. iCycler iQ PCR plates (Bio‐Rad Laboratories) were sealed with iCycler iQ Optical Quality Sealing Tape (Bio‐Rad Laboratories), and samples were run in triplicates in the following mode: 10 min at 95°C followed by 40 cycles of 35 s at 95°C and 45 s at the primer‐specific temperature. Relative quantification of chemokine receptor gene expression was carried out by normalization to the reference gene GAPDH (glyceraldehyde 3‐phosphate dehydrogenase). Senescence markers on gene expression level were analyzed using TaqMan Gene Expression Assay (Thermo Fisher Scientific) according to the manufacturer’s instructions. Briefly, samples were run in triplicates at 20 μL per well, consisting of UltraPure distilled water (Thermo Fisher Scientific), 20× TaqMan Gene Expression Assay (refer to Supporting Information 3 for Gene Assay ID), 2× TaqMan Gene Expression Master Mix (both Thermo Fisher Scientific), and 1 μL cDNA. MicroAmp Fast 96‐Well Reaction Plates (0.1 mL) (Thermo Fisher Scientific) were sealed with an optical quality sealing tape (Bio‐Rad Laboratories) and ran in the StepOneTM Real‐Time PCR System (Applied Biosystems) according to the manufacturer’s instructions.

PPIA and GAPDH were used as reference genes, and the Pfaffl [55] method was applied for analysis. Comparison between ND and OA MSCs was subsequently carried out by referring the normalized values of each OA sample to the average of the three ND samples.

### 2.9. *β*‐gal Staining

The histochemical staining of *β*‐gal was done according to the kit manufacturer’s instructions (Sigma Aldrich). Cells were washed twice with PBS 48 h after seeding in quadruplicates in a 12‐well format, fixated with fixation buffer for 6 min, washed three times with PBS, and incubated with the staining mixture for 7 h. Subsequently, the staining solution was removed, and cells were stained with DAPI (BD Pharmingen, 1:1000 in PBS). The histochemical staining of *β*‐gal was captured in bright field and DAPI under the fluorescence microscope. Both picture entities were merged to improve cell identification. Cells stained blue were counted as *β*‐gal‐positive, and their number was compared to the absolute number of cells, which were assessed by the nuclei stained with DAPI. Blinded assessment was performed by encrypting the file names using a plug‐in from ImageJ.

### 2.10. Analysis of Cell Culture Supernatant

Cell culture supernatant from the samples used for RNA isolation was employed for the subsequent experiment. The medium was removed from cells and stored at −20°C until further use. For the analysis the Legendplex Human Inflammation Panel 1 (Biolegend, San Diego, CA, USA) was used, which includes the following chemokines and cytokines: IL‐1*β*, interferion (IFN)‐*α*2, IFN‐*γ*, TNF‐*α*, monocyte chemoattractant protein‐1 (MCP‐1) (CCL2), IL‐6, IL‐8 (CXCL8), IL‐10, IL‐12p70, IL‐17A, IL‐18, IL‐23, and IL‐33. The procedure was performed according to the kit’s protocol in the recommended duplicates using V‐bottom plates. Wells were loaded with assay buffer and sample or standard, and incubated for 2 h at room temperature on a plate shaker at 800 rpm. The plate was centrifuged for 5 min at 250 *g*, the supernatant was removed, wells were washed with wash buffer, and the detection antibody was added and incubated for 1 h at room temperature on the plate shaker. SA‐PE was added and once again incubated for 30 min. Afterward, wells were washed twice, and the beads were resuspended in wash buffer. Samples were read on a FACSCantoII Flow Cytometer, and data analysis was performed using the included software. Estimated concentrations are based on an 8‐point standard curve. The same medium cultivated without cells for the same period of time served as negative control.

### 2.11. Statistical Analysis

All experiments were carried out with three individual donors per experimental group. Student’s *t*‐test was applied to determine significant differences. Results with significant *p*‐values are denoted as follows:  ^∗^ *p*  < 0.05, 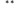 *p* < 0.01, ^#^ *p* < 0.001. Data are shown as mean, and error bars indicate standard deviation. Data analysis and design of graphs were performed using GraphPad Prism Version 9.0.

## 3. Results

### 3.1. ND and OA MSCs Differentiate Toward the Osteogenic, Adipogenic, and Chondrogenic Lineage

MSCs derived from both sources adhered to plastic surfaces and maintained a spindle‐shaped morphology throughout cultivation with a tendency toward a more stretched form with increasing Passage (Figure 1A). MSCs from ND and OA donors exhibited comparable growth kinetics between Passages 1 and 4 (Supporting Information 4). Mean values of the specific growth rate *μ* were 0.099 ± 0.03 for ND and 0.13 ± 0.04 for OA cultures. Although OA MSCs displayed a slightly higher proliferative activity, particularly after Passage 3, this difference did not reach statistical significance. Expression of surface markers was assessed for Passage 3 ND and OA MSCs using flow cytometry. Cells of both groups displayed CD44, CD73, CD90, CD105, and CD166 and lacked expression of CD14, CD34, and CD45 (Supporting Information 5). ND and OA MSCs were evaluated for their ability to differentiate toward the adipogenic, osteogenic, and chondrogenic lineages. After adipogenous induction for 15 days, all samples formed lipid vacuoles as demonstrated by Oil Red O staining (Figure 1A). Osteogenic differentiation was determined by von Kossa and AP staining after 28 days. Von Kossa staining revealed a donor‐dependent mineralization in both groups, while AP activity was detected consistently (Figure 1A). Semiquantitative assessment of Oil Red O, AP, and von Kossa is available as Supporting Information 6.

Figure 1Adipogenic, osteogenic, and chondrogenic differentiation of ND and OA MSCs. Both OA and ND MSCs showed a fibroblast‐like morphology in monolayer cell culture and were subsequently induced to differentiate toward the adipogenic, osteogenic, and chondrogenic lineages. (A) Oil Red O staining indicates successful lipid vacuole formation, and Von Kossa and AP staining demonstrate mineralization and AP activity. (B) Safranin O and Alcian Blue were used to visualize proteoglycans and mucopolysaccharides, and collagen type 2 was assessed immunohistochemically; the negative control is depicted in the top left corner. No difference in differentiation capacity between ND and OA MSCs was observed. (C) Quantification of proteoglycans stained by Safranin O was conducted using Bern Score, revealing a comparable result between ND and OA MSCs (8.13 vs. 7.89).

**Figure:**
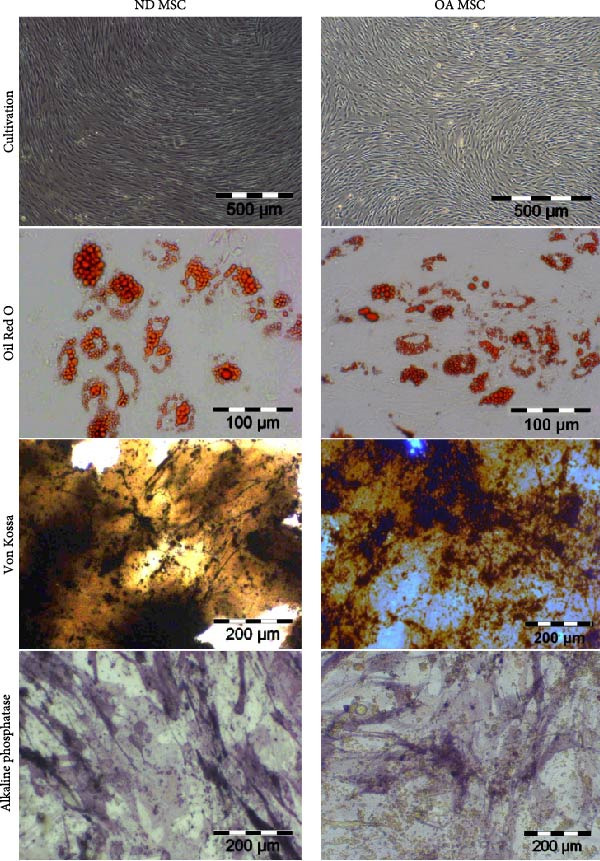
(A)

**Figure:**
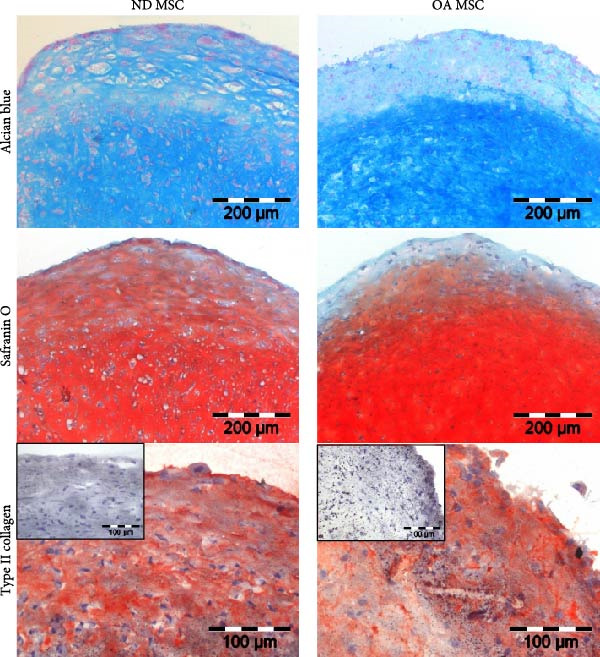
(B)

**Figure:**
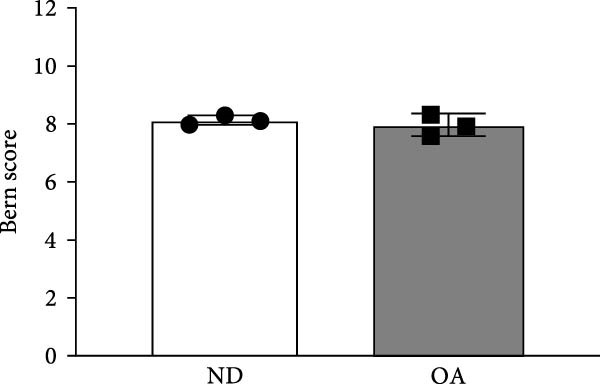
(C)

Passage 3 ND and OA MSCs were stimulated in a high‐density pellet culture for 28 days to analyze chondrogenic differentiation potential. Positive Safranin O and Alcian blue 8GX staining confirmed the presence of proteoglycans and mucopolysaccharides, while immunohistochemistry attested to the expression of type II collagen (Figure 1B). All pellets met these two criteria of chondrogenic differentiation. Bern score was determined to quantify and compare cartilage formation (Figure 1C). Mean Bern score values of 8.13 ± 0.16 and 7.89 ± 0.38 for ND and OA MSCs, respectively, indicate comparable chondrogenic differentiation potential (*p* = 0.38). In summary, these results reveal that MSCs of both ND and OA donors are capable of multilineage differentiation.

### 3.2. Features of Cellular Senescence Do Not Significantly Differ Between ND and OA MSCs

To compare senescence levels between ND and OA MSCs, the activity of *β*‐gal was evaluated histochemically (Figure 2A, B). RT‐qPCR was used to evaluate the expression of cyclin‐dependent kinase (CDKN) inhibitor 1A (CDKN1A), also known as p21^Waf1/Cip1^, CDKN inhibitor 2A (CDKN2A), also known as p16^INK4a^, matrix metallopeptidase 1 (MMP1), and sirtuin 1 (SIRT1) (Figure 2C). OA MSCs contained a higher percentage of *β*‐gal positive cells—31% compared to 24% in ND MSCs (Figure 2B); however, this outcome did not amount to a statistically significant difference (*p* = 0.5). The share of *β*‐gal positive cells varied greatly between donors, ranging from 20% to 37% and 14% to 39% in OA and ND donors, respectively. On the gene expression level, none of the selected senescence markers differed significantly between OA and ND MSCs. p21^Waf1/Cip1^ and SIRT1 showed a log_2_ fold change of −0.02 and −0.32 in OA MSCs, respectively, compared to the mean of ND MSCs. The expression of p16^INK4a^ in OA MSCs was continuously elevated with a mean log_2_ fold change of 1.3 compared to ND MSCs (*p* = 0.09). Although the mean expression of MMP1 was comparable between ND and OA, it is the gene with the greatest variability in the OA group, ranging from a log_2_ fold change of −6.6 to 3.1. Thirteen cytokines and chemokines were measured by LEGENDplex bead‐based immunoassay in the cell culture supernatant to account for a share of the SASP. The concentration of IL‐1*β*, IFN‐*γ*, tumor necrosis factor alpha (TNF*α*), IL‐10, IL‐12p70, IL‐17A, IL‐23, and IL‐33 remained below detection level in all groups and thus could not be determined. Figure 2D shows the results for the detected chemokines and cytokines. IFN‐*α*2, MCP‐1, and IL‐6 did not differ significantly between ND and OA MSCs. The amount of secreted IL‐6 appears to be donor‐dependent, ranging from 1125 to 1422 pg/mL in OA and ND MSCs, respectively. IL‐18 was significantly lower in OA than in ND MSCs (4.9 vs. 17 pg/mL). One value stayed below the detection level of 3.94 pg/mL. This numerical threshold for detection was used for graphical and statistical analysis. IL‐8 was secreted significantly higher in ND than in OA MSCs (3764 vs. 555 pg/mL).

Figure 2Assessment of senescence by analysis of *β*‐Galactosidase, the senescence markers CDKN1A, CDKN2A, MMP1, and SIRT1, and the SASP. ND and OA MSCs were stained for *β*‐Galactosidase activity, and RT‐qPCR was performed to measure the expression level of selected genes. A LEGENDplex bead‐based immunoassay was used to examine the concentration of selected cytokines and chemokines in cell culture medium. (A, B) Blue cells in bright field were counted as *β*‐Galactosidase positive and divided by the total number of cells indicated by DAPI‐stained nuclei. ND and OA MSCs exhibit a share of 24% ± 11% and 31% ± 7% *β*‐Galactosidase positive cells, respectively. (C) Selected senescence markers were normalized to the reference genes GAPDH and PPIA. Boxes represent the log_2_ fold change of individual donors and are relative to the mean of ND. No significant difference in gene expression was observed between ND and OA MSCs. (D) IL‐18, MCP‐1, IFN‐*α*2, IL‐8, and IL‐6 were measured above detection level, and a significant increase in ND compared to OA MSCs was found for IL‐18 (17 vs. 5 pg/mL) and IL‐8 (3764 vs. 555 pg/mL). Culture medium without cells incubated for the same period served as negative control.  ^∗^ *p*  < 0.05,  ^∗∗^ *p*  < 0.01.

**Figure:**
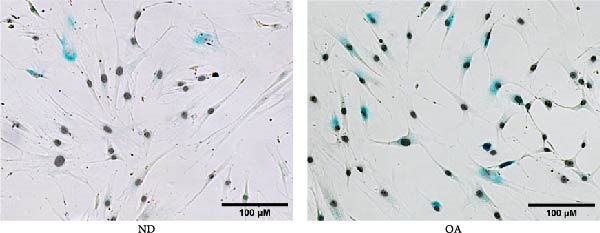
(A)

**Figure:**
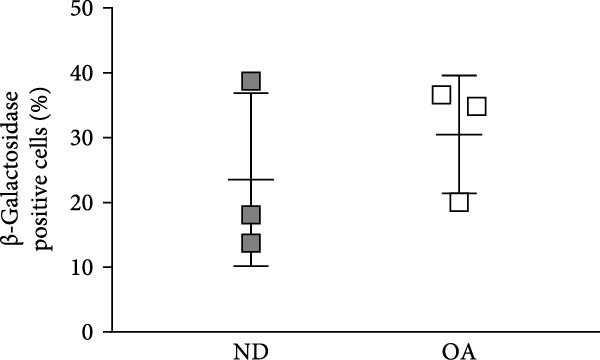
(B)

**Figure:**
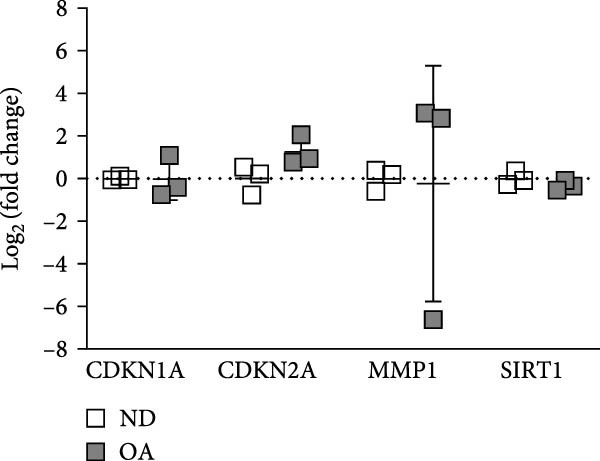
(C)

**Figure:**
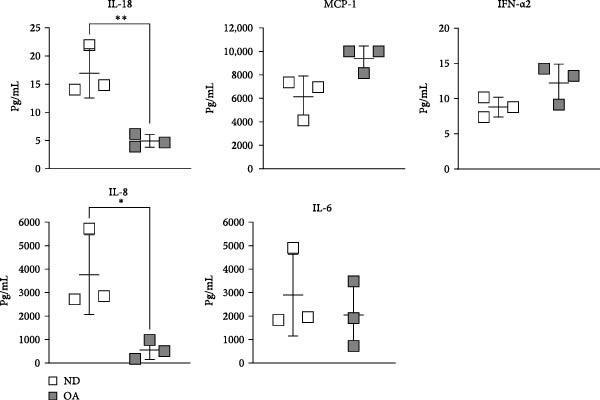
(D)

### 3.3. Genes Associated With Senescence Reveal a Similar Expression Pattern in ND and OA MSCs

**Figure 3:**
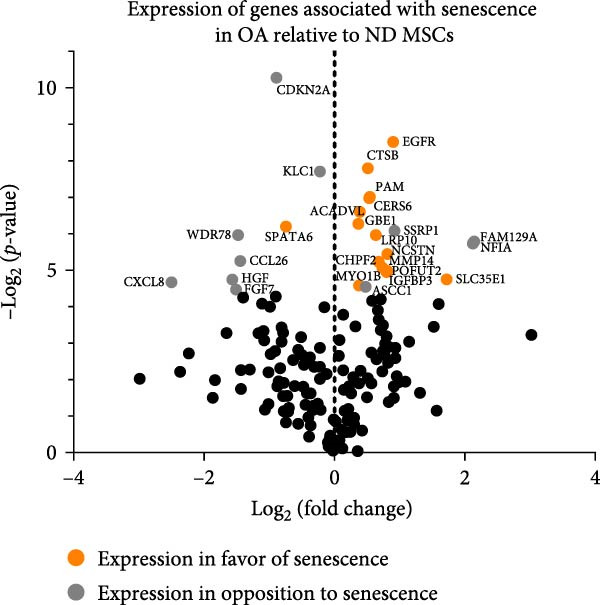
Comparison of the expression of genes associated with senescence between ND and OA MSCs. In total, 154 genes associated with senescence were analyzed regarding their differential expression in OA compared to ND MSCs in a microarray dataset. The log_2_ fold change refers to the fold expression of the respective gene in OA compared to ND MSCs. Significantly differentially expressed genes in OA MSCs are depicted in gray and orange, accompanied by their gene symbol.

Since senescence‐dependent gene expression may negatively influence the regenerative potential of MSCs, a selective analysis of 187 genes associated with senescence (Supporting Information 2) was performed using an RNA microarray dataset of ND and OA MSCs (Figure 3). In total, 154 genes were significantly detected and used for further analysis. The log_2_ fold change refers to the change of gene expression in OA compared to ND MSCs. Significantly DEGs are colored. Orange illustrates a gene expression pattern that would be expected in senescent cells, while gray indicates genes where a senescence phenotype is less likely. In total, 26 senescence‐associated genes are significantly differentially expressed. Among the 15 genes associated with a senescent phenotype, 14 exhibit less than a two‐fold change in differential expression. Only solute carrier family 35 member E1 (SLC35E) shows a 3.3‐fold upregulation. Moreover, nine of these genes (SPATA6, PAM, ACADVL, GBE1, LRP10, NCSTN, POFUT2, CHPF2, SLC35E1) are associated with senescence, but not directly involved in its functional mechanisms [52–54]. In contrast, five of these genes (EGFR, CTSB, CERS6, MMP14, MYO1B, IGFBP3) have been directly implicated in the regulation or progression of senescence [37, 39, 56–58]. In total, 11 genes reveal an expression pattern that would not indicate an increased senescence level. Among them are IL‐8, also known as CXCL8, and CDKN2A, which are two prevalent markers of senescence and show a 5.7‐ and 1.9‐fold downregulation, respectively. While the differential expression of CDKN2A could not be confirmed by RT‐qPCR (Figure 2C), secretion of CXCL8 was significantly lower in the cell culture supernatant of OA compared to ND MSCs (Figure 2D). This analysis reveals that the majority of genes related to senescence, namely 128 out of 154 genes, exhibit no significant alteration between OA and ND MSCs. In conclusion, OA MSCs are comparable to ND MSCs in terms of their multilineage potential, senescent phenotype, and expression of genes associated with senescence.

### 3.4. Both ND and OA MSCs Express a Majority of Chemokine Receptors on the Gene and Protein Level and Migrate Toward CCL25

Since cell migration mediated by chemokines might pose a valuable approach in the field of in situ tissue engineering, the chemokine receptor profile of OA MSCs using RT‐qPCR and immunohistochemical staining was examined (Figure 4A, B). The results were subsequently compared to the chemokine receptor profile of ND MSCs, which was published by our group previously [46] (Figure 4B). CCR6, CCR7, CCR9, and CXCR2‐6 were present in all OA MSCs on gene expression and protein levels. CCR1, CCR3, CCR5, and CXCR1 showed a donor‐dependent expression on the protein level, while for CCR2 and CCR4, this is true on the protein and gene expression levels. CCR8, CCR10, and CX3CR1 were continuously detected via RT‐qPCR but not on the protein level. ND MSCs steadily express all chemokine receptors on the mRNA level and CCR8, CCR9, and CXCR1‐CXCR6 on the protein level. CCR1‐7, CCR10, and CX3CR1 displayed donor‐dependent expression, and no expression was detected for CCR4. Out of all examined chemokine receptors, CCR9 and CXCR2‐CXCR6 showed a steady expression on gene expression and protein levels in OA and ND MSCs, proving to be an adequate target for chemokine‐mediated in situ tissue engineering.

Figure 4Analysis of the chemokine receptor profile of OA MSCs on gene expression and protein level and comparison to previously published data from ND MSCs [46]. (A) RT‐qPCR demonstrates a donor‐independent presence of CCR1, CCR3, CCR5‐10, CXCR1‐6, CX3CR1, and XCR1 in OA MSCs. Values are shown as percentage of GAPDH expression. (B) Immunohistochemical staining of chemokine receptors in OA MSCs reveals a continuous expression of CCR6, CCR7, CCR9, and CXCR2‐CXCR6. All chemokine receptors are present in ND MSCs on gene expression level, while it is donor‐dependent for CCR1‐3, CCR5‐7, CCR10, and CX3CR1 on protein level and could not be confirmed at all for CCR4. (+, expression in all three donors; −, no expression in all three donors; (+), donor‐dependent expression; n.d., not determined).

**Figure:**
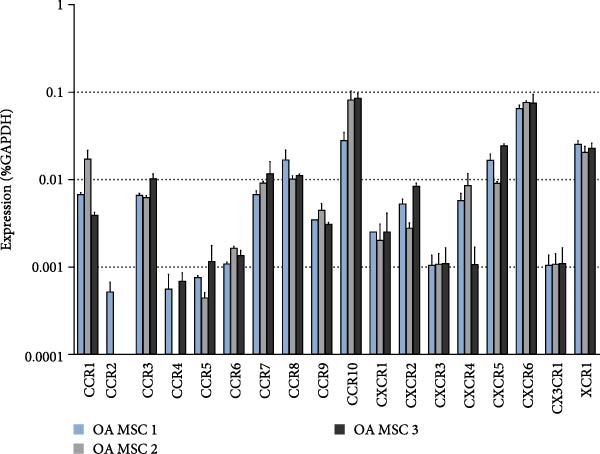
(A)

**Figure:**
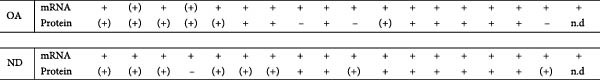
(B)

Once the trilineage differentiation and the expression of chemokine receptors essential for their migratory potential were validated and a prevailing senescence phenotype was excluded, we sought to examine the migratory ability of MSCs. We hereby focused on CCL25 as a chemoattractant due to its reliable CCR9 expression and prior results suggesting its chemotaxis‐enhancing capacity [26–28]. Neither ND nor OA MSCs migrated toward CCL25 at a concentration of 1, 10, or 100 nM in DMEM containing 0.1% FBS (Figure 5A, B). At a concentration of 1000 nM, both OA and ND MSCs showed significant migration toward CCL25 (Figure 5A, B), as indicated relative to the negative control (DMEM containing 0.1% FBS). Notably, more cells in the ND group migrated toward CCL25, as represented by the chemotactic index for ND MSCs (2, 5.9, and 6.4) compared to OA MSCs (1.9, 2.4, and 3.9). However, this difference does not reach statistical significance (*p* = 0.25). Hence, CCL25 at 1000 nM leads to a stable and significant migration of OA MSCs. This concentration was subsequently used to study the effects of CCL25 on OA MSCs on gene expression levels.

Figure 5Migration of ND and OA MSCs toward CCL25. ND and OA MSCs were treated with CCL25 at the indicated concentrations, and migrated cells were counted after 20 h. The chemotactic index represents the ratio of migrated cells to the negative control (0 nM CCL25, 0.1% FBS) for each concentration. Significant migration was observed for ND (A) as well as OA MSCs (B) at 1000 nM CCL25.  ^∗^ *p*  < 0.05,  ^∗∗^ *p*  < 0.01, ^#^ *p*  < 0.001.

**Figure:**
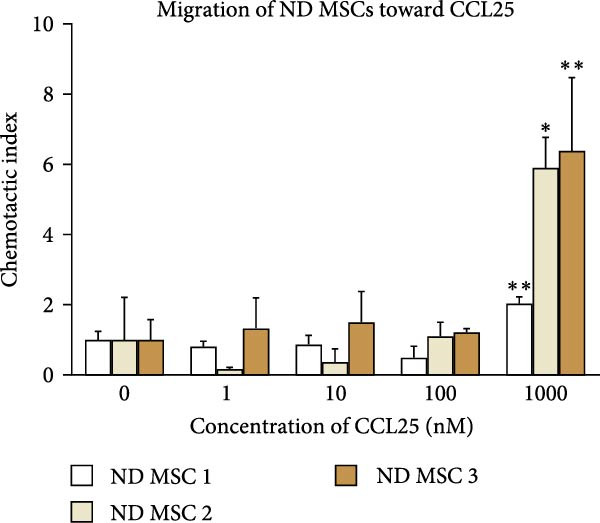
(A)

**Figure:**
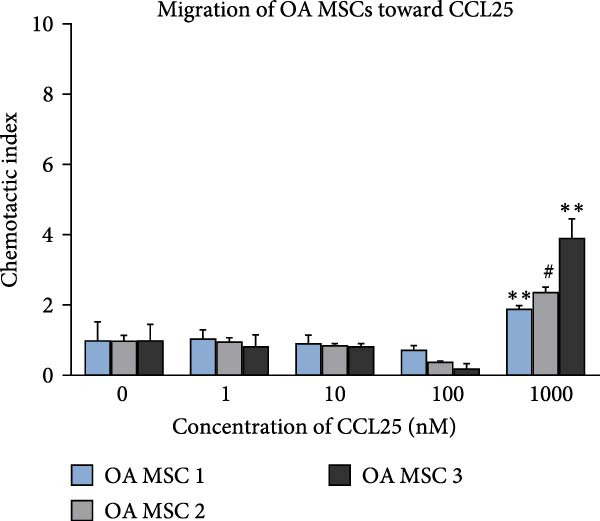
(B)

Proliferation and apoptosis of OA MSCs treated with 1000 nM CCL25 for 20 h were analyzed to rule out potential harmful effects on cells. Proliferative capacity was determined for 2 weeks and showed a donor‐dependent divergence, but no significant change in response to CCL25, as calculated by the PD. PD for cells treated with 1000 nM CCL25 was on average 2.3 (±0.03) and for control cells 2.35 (±0.2) (Figure 6A). Apoptosis was assessed immediately after CCL25 treatment. While the positive control exhibits an average of 36.4% of apoptotic and dead cells as determined by annexin V and PI staining, this share was 2.5% and 2.0% for CCL25‐treated cells and the negative control, respectively (Figure 6B). Hence, 1000 nM CCL25 neither halts nor promotes proliferation and does not induce apoptosis in OA MSCs within the observed time frame.

Figure 6Proliferation and apoptosis of OA MSCs in response to 1000 nM CCL25. OA MSCs were treated with 1000 nM CCL25 in deprivation medium for 20 h. Proliferation (A) was assessed by reseeding cells after 20 h of CCL25 treatment (day 1) and subsequently culturing them until confluency, counting them, and calculating the population doubling, which amounted to 2.3 and 2.35 over a time period of 13 days for the 1000 nM CCL25‐ and the deprivation medium‐treated cells, respectively (*p* = 0.9). Apoptosis and cell death (B) were determined by Annexin V and PI using flow cytometry. Cells cultured in deprivation only served as negative control and MSCs incubated at 55°C for 30 min as positive control. The latter demonstrated a significantly greater share of Annexin V‐ and PI‐positive cells compared to the CCL25‐treated cells (*p*  < 0.05), while no significant difference was found between the CCL25‐treated and the negative control group (*p* = 0.43).

**Figure:**
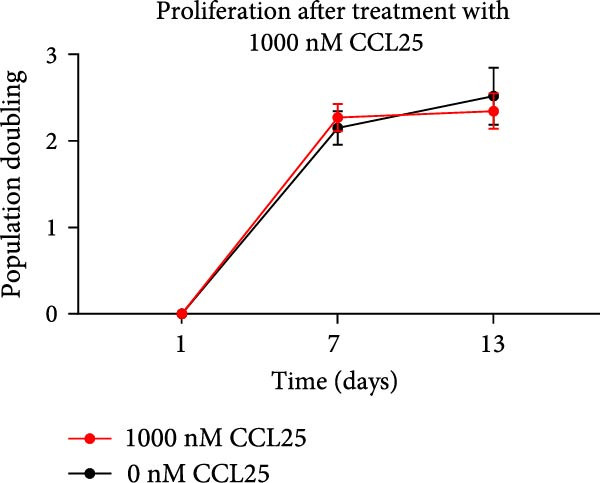
(A)

**Figure:**
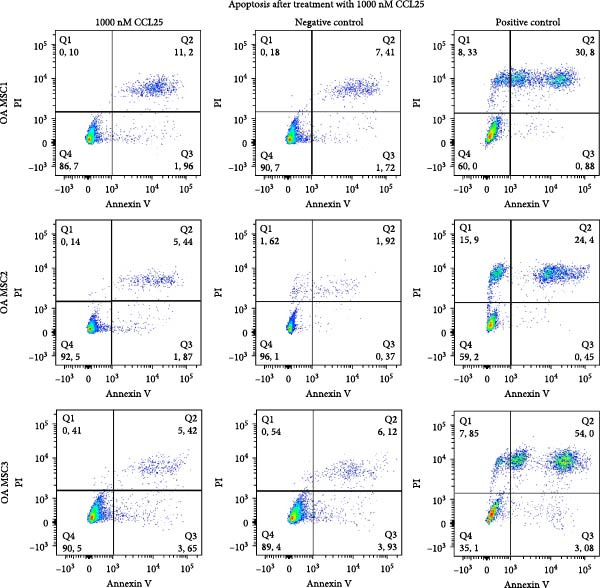
(B)

To investigate the broader effect CCL25 exerts on OA MSCs, gene expression profiles of OA MSCs with and without stimulation with 1000 nM CCL25 were compared using a genome‐wide microarray analysis. Significantly DEGs with a fold change greater than 2 or less than −2 were analyzed, yielding 777 DEGs (Supporting Information 7) out of 54,675 probe set IDs. Of these, 358 were upregulated and 419 downregulated. Enriched GO terms and KEGG pathways (Supporting Information 8) were subsequently examined to summarize the biological implications of CCL25 treatment.

### 3.5. CCL25 Exerts a Substantial Effect on Genes Associated With Migration

The 10 most significantly enriched GO terms out of 91 are pictured in Figure 7A. Table 1 lists these GO terms and their respective genes. Three terms in the top 10 directly indicate an effect on migration: regulation of cell migration (GO:0030334), which is the most significantly enriched GO term; positive regulation of cell motility (GO:2000147); and positive regulation of cell migration (GO:0030335). Moreover, skin development (GO:0043588) and collagen fibril organization (GO:0030199) are also related to homing of cells. Regulation of cell population proliferation (GO:0042127) contains the most genes with 56, followed by positive regulation of cellular processes (GO:0048522) with 46, and positive regulation of cell population proliferation (GO:0008284), and regulation of cell migration (GO:0030334) with 41 genes each. Skin development (GO:0043588), positive regulation of extrinsic apoptotic signaling pathway (GO:2001238), L‐alpha‐amino acid transmembrane transport (GO:1902475), and collagen fibril organization (GO:0030199) consist of a relatively small number of genes, with 14, 9, 10, and 10 genes, respectively. At the same time, these are also the terms with the highest odds ratios, at 6.5, 10.2, 8.3, and 7.8.

Figure 7Enriched GO terms and their assigned categories. (A) Bubble size indicates the number of genes belonging to a GO term, and the color represents the significance level, with red being the most significant. (B) All 91 significantly enriched GO terms were assigned to 1 out of 15 categories, and these are shown according to their number of genes and GO terms. More than four‐fold up‐ (C) or downregulated (D) genes after treatment of OA MSCs with 1000 nM CCL25 are depicted according to their category and their differential expression, indicated by log_2_ fold change.

**Figure:**
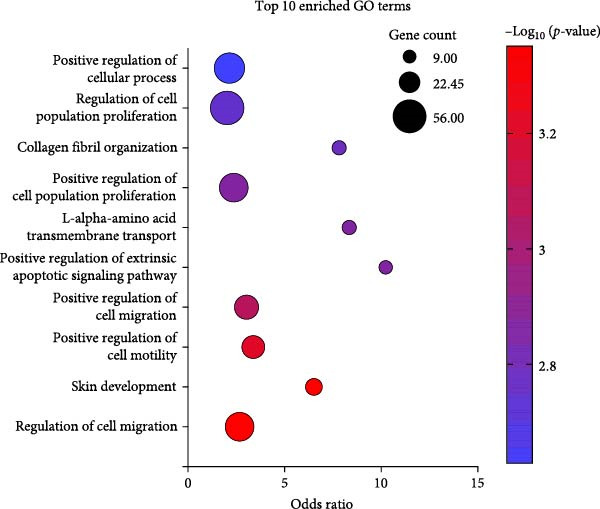
(A)

**Figure:**
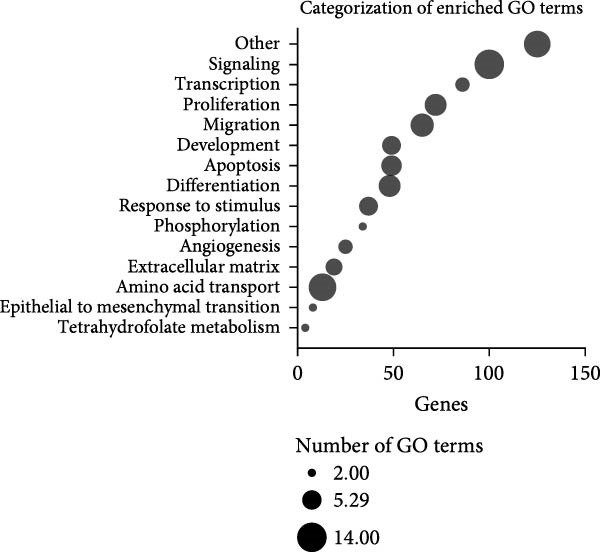
(B)

**Figure:**
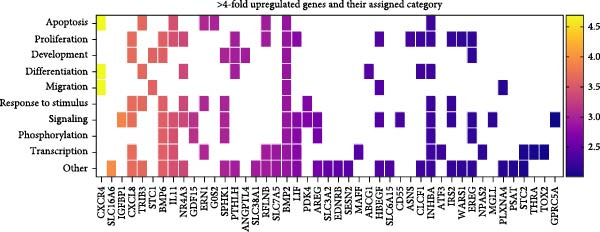
(C)

**Figure:**
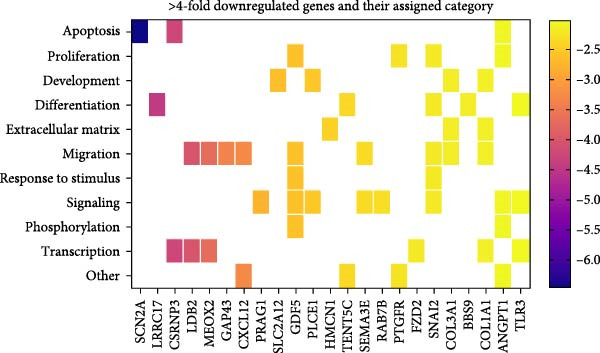
(D)

**Table 1:** Top 10 enriched GO terms and their associated genes.

GO term	Associated genes
Regulation of cell migration (GO:0030334)	BMP2, CDH11, CLDN4, COL1A1, CXCL12, CXCR4, DOCK10, EMILIN1, FGF2, GPER1, HBEGF, HGF, IGFBP5, INSR, LDB2, MMP14, MYLK, NEDD9, NEXN, PDGFA, PDGFRA, PLXNA4, RAP2B, RECK, RHOB, SEMA3C, SEMA3E, SEMA4C, SEMA5A, SERPINE1, SINHCAF, SNAI2, SPHK1, SPRY2, STC1, STK24, TCAF1, TIAM1, TWIST1, VEGFA, VSIR
Skin development (GO:0043588)	AKR1C3, BCR, CFLAR, COL1A1, COL1A2, COL3A1, COL5A1, COL5A2, CSTA, DHCR24, EREG, PDGFA, PKD1, UGCG
Positive regulation of cell motility (GO:2000147)	BMP2, CLDN4, COL1A1, CXCL12, CXCR4, GPER1, HBEGF, HGF, INSR, MMP14, MYLK, NEDD9, NTN1, PDGFA, PDGFRA, SEMA3C, SEMA3E, SEMA4C, SEMA5A, SNAI2, SPHK1, SPRY2, TIAM1, TWIST1, VEGFA, VSIR
Positive regulation of cell migration (GO:0030335)	AGO2, ARHGEF2, BMP2, CCN4, CLDN4, COL1A1, CXCL12, CXCR4, GPER1, HBEGF, HGF, INSR, MMP14, MYLK, NEDD9, PDGFA, PDGFRA, SEMA3C, SEMA3E, SEMA4C, SEMA5A, SNAI2, SPHK1, SPRY2, TGFB2, TIAM1, TWIST1, VEGFA, VSIR
Positive regulation of extrinsic apoptotic signaling pathway (GO:2001238)	BID, G0S2, GPER1, INHBA, PPP2R1B, PYCARD, TNFRSF12A, TNFSF10, WWOX
L‐alpha‐amino acid transmembrane transport (GO:1902475)	SLC1A4, SLC25A12, SLC25A29, SLC38A1, SLC3A2, SLC66A1, SLC7A1, SLC7A5, SLC7A7, SLC7A8
Positive regulation of cell population proliferation (GO:0008284)	AKR1C3, AREG, BMP2, BMP6, CCN4, CDCA7L, CLCF1, CNOT7, EREG, FGF2, FOSL1, GAB2, GPER1, HBEGF, IL11, INSR, IRS2, LIF, MEGF10, MEIS2, NAMPT, NKX3‐1, NOP2, NR4A3, OSMR, PDGFA, PDGFRA, PTGFR, PTHLH, SINHCAF, SLC25A33, SPHK1, SSR1, TBC1D8, TBRG4, TGFB2, TGFBR3, TIAM1, TMEM119, TSLP, VEGFA
Collagen fibril organization (GO:0030199)	ADAMTS2, COL1A1, COL1A2, COL3A1, COL5A1, COL5A2, DPT, LOXL1, LOXL3, TGFB2
Regulation of cell population proliferation (GO:0042127)	ADGRG1, AKR1C3, AREG, ARHGEF2, ATF5, BMP2, BMP6, CDCA7L, CDKN2C, CEBPA, CLCF1, CNOT7, CXCL8, EMILIN1, EREG, FGF2, FOSL1, GAB2, GPER1, HBEGF, IL11, INHBA, INSR, IRF1, IRS2, KAT2B, KLF11, KSR1, LDOC1, LIF, MEIS2, NAMPT, NKX3‐1, NOP2, NR4A3, NUDT6, NUPR1, OSMR, PDGFA, PDGFRA, PTGFR, PTHLH, SH2B3, SINHCAF, SLC25A33, SSR1, TBC1D8, TBRG1, TBRG4, TGFB2, TIAM1, TSLP, UHRF1, ULK1, VEGFA, WARS1
Positive regulation of cellular process (GO:0048522)	ADGRG1, AKR1C3, AREG, BMP2, BMP6, CDCA7L, CHEK1, CLCF1, CNOT7, CXCL12, EGR2, EREG, FGF2, FOSL1, GAB2, GPER1, HBEGF, IL11, INSR, IRS2, LIF, MEIS2, MMP14, NAMPT, NEDD9, NKX3‐1, NOP2, NTN1, NUPR1, OSMR, PDGFA, PDGFRA, PTGFR, PTHLH, SINHCAF, SLC25A33, SPHK1, SSR1, TBC1D8, TBRG4, TGFB2, TIAM1, TNFRSF1B TSLP, TWIST1, VEGFA

All 91 significantly enriched GO terms were then classified into 15 categories (Figure 7B), primarily derived from their respective GO labeling. If the label did not coincide with the category, GO terms were matched to the most suitable category based on function. For example, positive regulation of cell population proliferation (GO:0008284) and positive regulation of cell cycle (GO:0045787) were both assigned to the category *proliferation*. The category *other* contains the most genes and GO terms, which can be mainly attributed to the fact that it also incorporates unspecific GO terms such as positive regulation of cellular processes (GO:0048522) and negative regulation of cellular processes (GO:0048523) (GO:0050866), consisting of 46 and 39 genes, respectively. *Signaling*, *transcription*, *proliferation*, and *migration* contain 100, 86, 72, and 65 genes, respectively. While *amino acid transport* contains only 13 genes, it is associated with the second‐most GO terms (12). The number of GO terms defining a category varies between 2 (*tetrahydrofolate metabolism*, *epithelial to mesenchymal transition*, *phosphorylation*) and 14 (*signaling*).

To shed light on the functional role of individual genes, more than four‐fold upregulated and downregulated genes were assigned to their attributed categories if they were previously associated with a GO term as defined by Enrichr (Figure 7C, D). For clarity, categories containing less than four genes in the upregulated and less than two genes in the downregulated group are not shown. A total of 81 genes were more than four‐fold upregulated (Figure 7C). Of those, 47 were assigned an enriched GO term. Apart from *others*, *signaling* is the category with the most genes, namely 18, followed by *transcription* and *proliferation* with 15 genes. *Response to stimulus*, which includes GO terms that are parent terms of chemotaxis, holds 10 genes, while *migration* holds five genes. CXCR4, the second‐highest expressed gene after stimulation with CCL25, is also involved in *differentiation* and *apoptosis*. The CXCR4/CXCL12 interaction is one of the best‐described signaling pathways in terms of cellular migration. While CXCR4 is upregulated, the expression of CXCL12 is more than 10‐fold downregulated (Figure 7D). The chemokine CXCL8 reveals an ~14‐fold upregulation due to CCL25 and an association with *proliferation*, *development*, *response to stimulus*, *signaling*, *transcription*, and *other* processes.

Of the 66 more than four‐fold downregulated genes, 23 were assigned a GO term (Figure 7D). The categories containing the most genes are *migration*, *signaling*, and *transcription*, with 9, 8, and 6 genes, respectively. The most downregulated genes are SCN2A, LRRC17, and CSRNP3, which are associated with *apoptosis*, *differentiation*, and *transcription*.

Of those more than four‐fold DEGs, 34 upregulated and 43 downregulated genes are not associated with a significantly enriched GO term. To contextualize their function, the GO terms for biological processes of each gene according to the QuickGO library were assigned to the previously established categories. PDE4B, CXCL3, DCLK1, and ARHGAP18 are the only genes associated with *migration*. Table 2 lists these genes, their log_2_ fold change, and their associated category.

**Table 2:** More than four‐fold upregulated or downregulated genes in OA MSCs treated with CCL25 that were not assigned to an enriched GO term are listed with their log_2_ fold change.

Gene	log_2_ (FC)	Associated category	Gene	log_2_ (FC)	Associated category	Gene	log_2_ (FC)	Associated category
C2CD4A	5.2	Other	CTH	2.6	Response to stimulus, apoptosis, signaling, differentiation	SNCAIP	2.2	Apoptosis
ATP6V0D2	4.3	Other	JARID2	2.6	Transcription, development, proliferation, differentiation, transcription	LOC102724560	2.1	—
SMIM32	4.0	—	VLDLR	2.5	Signaling, development	RASD1	2.1	Signaling, transcription
SMOX	3.7	Other	SOCS2‐AS1	2.5	—	MEDAG	2.1	Differentiation
BEX2	3.4	Signaling, apoptosis, proliferation	C17orf58	2.4	Extracellular matrix	LOC105371401	2.1	—
KCNG1	3.3	Other	IL18R1	2.4	Signaling, differentiation	ST3GAL1	2.1	Differentiation, extracellular matrix, apoptosis
ISG20	3.0	Transcription, response to stimulus	FAM167A	2.3	—	FST	2.1	Development, transcription, differentiation, signaling, proliferation
FAM124A	2.9	—	ITPRIP	2.3	Apoptosis	EFHD1	2.1	Development
PITPNC1	2.8	Signaling	**CXCL3**	**2.3**	**Migration**, response to stimulus	PAQR5	2.0	Differentiation
**PDE4B**	**2.8**	Signaling, **migration**, response to stimulus	RCAN1	2.3	Response to stimulus, signaling, development	PHGDH	2.0	Development, transcription
PPP1R14C	2.8	—	RAB33A	2.2	Signaling	—	—	—
CHST7	2.7	Extracellular matrix	C2CD2	2.2	—	—	—	—
PLCB4	−3.8	Signaling	ST8SIA1	−2.7	Proliferation	PPM1H	−2.2	—
SETBP1‐DT	−3.6	—	GNG2	−2.6	Signaling, proliferation, response to stimulus	PRSS23	−2.2	Other
ANK3	−3.5	Proliferation, signaling, development, transcription, response to stimulus	PDE5A	−2.6	Signaling, proliferation, development	LINC02458	−2.2	—
DDIT4L	−3.3	Signaling	AKR1B10	−2.4	Other	RAB30	−2.2	Signaling
COL21A1	−3.3	—	S100A3	−2.4	—	PDGFRL	−2.1	Signaling
TMT1A	−3.0	Differentiation	FIGN	−2.4	Proliferation	STXBP6	−2.1	Other
NAP1L3	−3.0	Other	TCF19	−2.4	Transcription	AGBL5	−2.0	Other
LINC02984	−3.0	—	PLCL1	−2.4	Signaling	HCFC1R1	−2.0	—
MXRA5	−3.0	Response to stimulus	**DCLK1**	**−2.3**	**Migration**, development, differentiation, signaling	CRISPLD1	−2.0	Other
RCAN2	−2.9	Signaling	SETBP1	−2.3	Transcription	ACAT2	−2.0	Other
HELLS	−2.9	Development, apoptosis, transcription, proliferation	TCEA3	−2.3	Transcription	MEST	−2.0	Development
ARHGAP28	−2.8	Signaling	SYTL2	−2.3	Other	CCDC89	−2.0	—
SERTAD4	−2.8	—	**ARHGAP18**	**−2.3**	Signaling, **migration**	LRIG3	−2.0	Other
GASK1B	−2.7	—	CRYZL2P	−2.2	—	—	—	—
LINC02511	−2.7	—	FAM111A‐DT	−2.2	—	—	—	—

*Note:* Associated GO terms of each gene were assigned to the previously established categories. The field was left blank when no GO term for biological processes was found. Genes falling in the category migration are printed in bold letters.

In summary, CCL25 exerts a broad effect on OA MSCs, ranging from categories of miscellaneous implications like *signaling*, *transcription*, and *phosphorylation* to categories that are crucial for potential regenerative processes like *proliferation*, *migration*, and *development*, as well as *apoptosis*, *differentiation*, *angiogenesis*, and *extracellular matrix*.

### 3.6. The Cytokine–Cytokine Receptor Interaction Pathway Is Highly Enriched in OA MSCs Treated With CCL25

Twenty‐one KEGG pathways displayed significant enrichment when analyzing upregulated genes in MSCs stimulated with CCL25 compared to nonstimulated cells, whereas downregulated genes did not lead to a significantly enriched pathway. The most significantly enriched KEGG pathways were TGF‐*β* signaling pathway, cytokine–cytokine receptor interaction, and TNF signaling pathway, containing 11, 18, and 9 genes, respectively (Figure 8A). These pathways regulate a myriad of cellular functions, including proliferation, apoptosis, differentiation, inflammation, and migration. This is also true for the PI3K‐Akt signaling pathway and MAPK signaling pathway. Furthermore, pathways with metabolic implications, including the AMPK signaling pathway, are significantly enriched. Genes that were upregulated by more than four‐fold are highlighted in color based on their log_2_‐fold change (Figure 8B). Each gene is also categorized according to its corresponding KEGG pathway.

Figure 8Enriched KEGG pathways and contributing genes. Twenty‐one pathways showed significant enrichment and are depicted in different colors and bubble sizes, indicating significance level and the number of genes (A). More than four‐fold upregulated genes are shown in color according to their log_2_ fold change and are attributed to their respective KEGG pathway (B).

**Figure:**
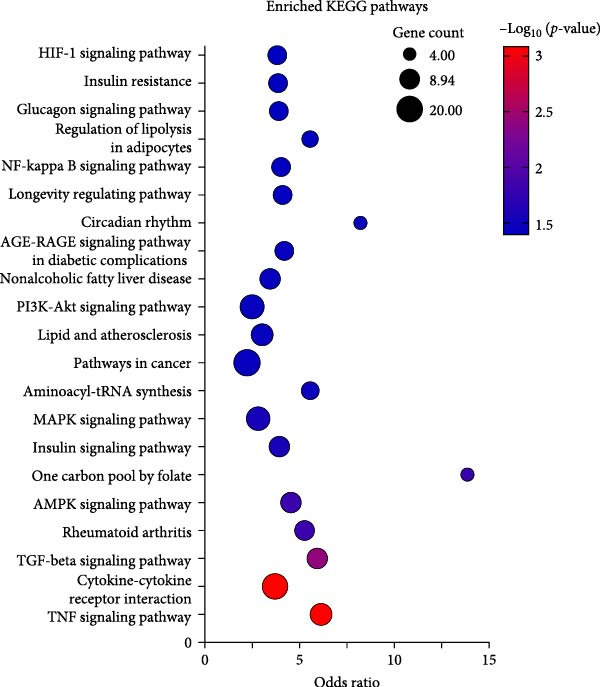
(A)

**Figure:**
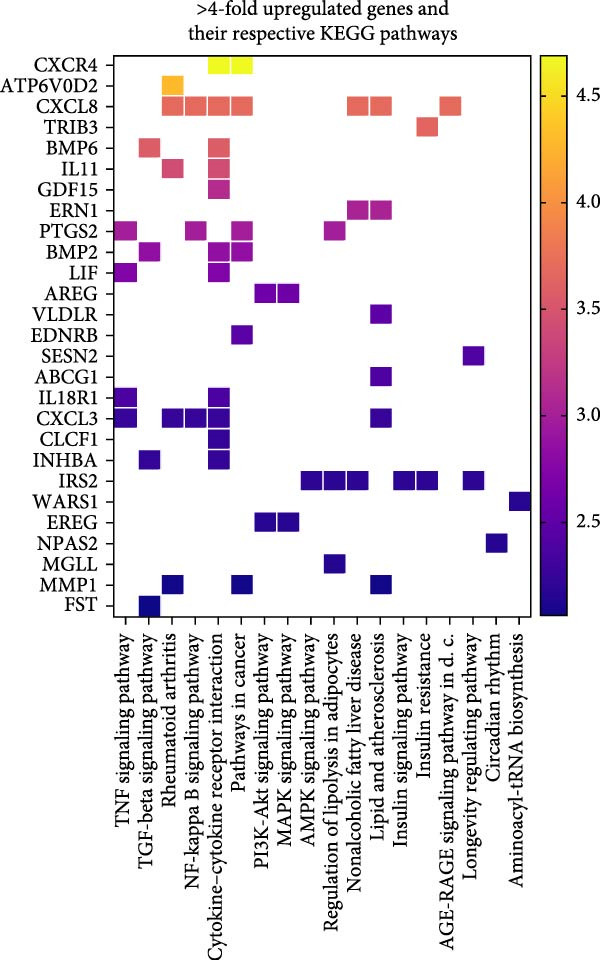
(B)

In summary, as already indicated by the enriched GO terms, CCL25 induces pathways involved in inflammatory and developmental processes, migration, metabolism, proliferation, and apoptosis.

## 4. Discussion

In this study, no considerable differences were observed between OA and ND MSCs in terms of differentiation capacity, senescence status, and chemokine receptor expression. These findings indicate that OA MSCs preserve the intrinsic regenerative competence and chemokine responsiveness required for drug‐based in situ tissue engineering, which highlights their suitability as an endogenous target cell population in OA. In addition, ND‐ and OA‐derived MSCs exhibited comparable growth kinetics, confirming similar proliferative potential under standardized culture conditions. The specific growth rates observed (~0.1 day^−1^) correspond to a PD time of about 1 week, consistent with published data for human BM‐MSCs [36].

Previous studies further comparing OA and ND MSCs have yielded conflicting results. To our knowledge, this is the first study to comprehensively assess all of these key functional features in parallel and to include a translational investigation of MSCs migration in OA, focusing on the chemokine CCL25. A subsequent genome‐wide analysis was conducted to explore the broader effects of CCL25 on OA MSCs, as previous observations are largely based on ND MSCs. Our findings revealed substantial alterations in genes associated with migration, differentiation, and metabolism. These findings provide an important foundation for advancing in situ tissue engineering strategies using OA‐derived MSCs, while also offering critical insight into the potential benefits and risks of employing chemokines like CCL25 in a translational context.

Previous works exploring potential differences between OA and ND MSCs have reported inconsistent findings. While some studies confirmed decreased proliferation [32] and chondrogenesis [32, 33], others did not find such a difference [36, 59]. Interestingly, the studies that did find a potential impairment of OA MSCs featured a considerably younger age range/mean age of ND compared to OA donors. Studies comparing MSCs based on age only demonstrated that MSCs derived from older donors exhibit reduced proliferation and osteogenesis [60], a higher level of senescent cells [60–62], and diminished regenerative [63] and chondrogenic differentiation [62] potential. One study did not find a difference between old and young MSCs regarding morphology, proliferation, and differentiation [64], but an impairment of these features following in vitro aging [64, 65]. The mean donor age in our study was 64 years for ND and 65 years for OA MSCs, concomitantly with sustained and comparable trilineage differentiation and no difference in senescence. These results indicate that the observed differences might be rather attributed to age than OA pathology. Excluding senescent MSCs in the context of in situ tissue engineering may indicate a greater likelihood of therapeutic success, as senescent MSCs might contribute to the progress of OA by secreting pro‐inflammatory and catabolic factors [40] or by exhibiting deteriorated immunomodulatory capacities [66] and might even be a causal contributor to the development of OA [42]. Additionally, outcomes of cell‐based tissue engineering approaches could be dampened by senescent MSCs. For example, the evidence of intra‐articular injection of MSCs is still ambiguous regarding their benefit in OA [67, 68]; ruling out senescence in the injected MSCs‐population could contribute to disambiguate these results.

The markers used to investigate senescence in this study did not yield a difference between ND and OA MSCs. Prominent members of the SASP, including IL‐1*β*, IL‐6, TNF‐*α*, CXCL8, and MCP‐1 [39], were not increased in the cell culture supernatant of OA compared to ND MSCs. Contrary to the hypothesis, CXCL8 was significantly downregulated in OA MSCs on gene expression level as measured by genome‐wide microarray analysis, and also on protein level, as measured in the cell culture supernatant. An increased level of CXCL8 in the serum and synovial fluid is associated with OA severity [69, 70], and a negative effect on chondrocytes in vitro was demonstrated previously [69, 71]. However, CXCL8 is a pleiotropic chemokine and has also been shown to foster cartilage regeneration in vivo [19, 72].

A limitation for this conclusion is the small number of samples. A broader spectrum of samples is needed, with biological age taken into account, to determine the role of senescence in MSCs from OA patients more clearly. Additionally, the question of the suitability of OA MSCs for in situ tissue engineering is tackled based on the three aspects of differentiation, senescent phenotype, and chemokine receptor profile. While these factors are crucial, more research, including functional assays as well as in vitro models that mimic an OA‐like environment, is necessary to determine this more thoroughly. Moreover, MSCs were derived only from bone marrow, and extraction from two different anatomical locations poses a limitation of this study. MSCs were characterized based on their adherence to plastic, trilineage differentiation, and MSC‐defining surface markers, which reflect the common approach for evaluating the general stromal population targeted by in situ tissue engineering. Although this strategy is translationally relevant because endogenous cell pools cannot be preselected in vivo, a more refined analysis of distinct MSC subsets may provide additional mechanistic insight. For example, CD271^+^ stromal progenitors exhibit enhanced skeletal lineage potential in certain contexts [73, 74], and OA bone morphology phenotypes such as hypertrophic remodeling may affect niche conditions and MSC behavior. Future work integrating subset‐specific characterization or stratification by OA bone phenotype could help to further optimize regenerative outcomes.

While studies have shown a potential systemic effect of OA on MSCs‐associated features like differentiation [32, 33], others indicate that the extent of the cartilage lesion does affect bone marrow‐derived MSCs [75] and that MSCs from bone marrow lesions display functional alterations [30]. In a potential avenue for future research, synovium‐derived MSCs are in close proximity to the damaged cartilage and are considered a promising source for cartilage regeneration [76, 77]. However, there is evidence that the OA microenvironment could impair stem cell function within the joint [78]. While the data regarding the impact of OA on MSCs are contradictory, an in situ tissue engineering approach taking advantage of multiple compounds that target not only migration but also, for example, differentiation and proliferation might be able to counteract potential aging‐related deficiencies. With the differentiation potential confirmed and a senescent phenotype excluded, the next step was to characterize the chemokine receptor profile of OA MSCs, given that chemokines not only recruit MSCs but may also enhance cartilage regeneration [19, 20, 29].

To our knowledge, this is the first study to characterize the chemokine receptor profile of MSCs derived from the bone marrow of OA donors. Previous studies have examined the chemokine receptor profile of MSCs isolated from healthy donors. Honczarenko et al. [79]. identified the surface expression of CCR1, CCR7, CCR9, and CXCR4‐CXCR6 but could not detect CCR2‐CCR6, CCR8, and CXCR1‐CXCR3. Experiments by our group subsequently revealed expression of these receptors as well [46]. When comparing the chemokine receptor profile of OA MSCs to data of ND MSCs collected by our group [46], the following stands out: The CXC‐receptors seem to be steadily expressed in ND and OA MSCs on protein and gene expression levels, except for CXCR1, which was detected only in two out of three OA donors on protein level. As for the CC‐receptors, CCR9 was the only receptor showing a steady expression in ND and OA MSCs on protein as well as on gene expression level. Hence, chemokines targeting either CXCR1‐CXCR6 or CCR9 might promote migration and potentially other regenerative properties with a higher probability than chemokines acting through CCR1‐8 or CX3CR.

Given the stable expression of CCR9 and the consistent migratory response of MSCs to 1000 nM CCL25, a genome‐wide transcriptomic analysis was conducted to assess the broader effects of CCL25 on OA MSCs. CCL25‐mediated homing has been previously described for subchondral progenitor cells at 1000 nM [80], for periosteum‐derived progenitor cells with a migration maximum at 500 nM [26], and for anulus fibrosus cells with a donor‐dependent migration maximum between 500 and 1000 nM [81]. Moreover, CCL25 was detected in synovial fluid at a low intensity, but a two‐fold increase in synovial fluid from OA compared to healthy donors was found [80]. Binger et al. [28] demonstrated the strong chemotactic effect of 1000 nM CCL25 on MSCs derived from healthy donors and examined its impact on genome‐wide gene expression. While genes involved in cellular movement were also increased in OA MSCs when treated with CCL25, there was no induction of CXCL1, CXCL2, and CXCL6 as found in ND MSCs, but only of CXCL8 and CXCL3. All of these chemokines activate CXCR1 and/or CXCR2. What limits this comparison is the diverging approach in terms of the statistical selection of genes and the different databases used for ontological gene assortment. In addition to its homing abilities, the effect of CCL25 on cartilage has been explored previously. In a 3D micromass model of porcine chondrocytes, Lüderitz et al. [82] found a negative effect of 500 nM of CCL25 on cartilage, indicated by decreased GAG, COL2A1, COL1A1, COL6A1, and increased MMP1. Concentrations between 0.05 and 50 nM did not have such an effect. Interestingly, increased MMPs (MMP1 and ‐14) and decreased collagens (COL21A1, COL3A1, COL1A1, COL1A2, COL8A1, COL5A1, and COL5A2) were also detected in OA MSCs treated with 1000 nM CCL25. Upregulation of genes involved in extracellular matrix degradation, alongside downregulation of matrix‐synthesizing genes, may impair cartilage formation but is likely crucial for enabling MSCs migration.

Given the pleiotropic nature of certain chemokines, it is essential to readdress the potential risks associated with their use. The CXCR4/CXCL12‐axis is well‐described in the context of attracting MSCs [20, 83, 84] and has even been shown to enhance cartilage formation [20]. However, evidence suggests that CXCL12 could as well contribute to cartilage degeneration [85, 86]. Our gene array data indicate an effect of CCL25 on apoptotic processes. Previous work demonstrated that viability in chondrocytes is not decreased when testing concentrations up to 500 nM of CCL25 [82]. Binger et al. [28] found 12 genes associated with apoptosis. Comparing these with the DEGs in our dataset, genes like GDF5 and TNFAIP3, which are involved in many cellular processes, represent the common denominator. In our analysis, 1000 nM CCL25 did not lead to an increased rate of apoptotic or dead cells (Figure 6B), nor did it impact the proliferation of OA MSCs. The fact that 1000 nM CCL25 does not lead to apoptosis in OA MSCs constitutes a relevant requisite for future research and in vivo models. While gene array data revealed an involvement of genes associated with apoptosis and proliferation, gene expression profiles can reflect early or subtle changes in gene activity that might not yet translate into measurable phenotypic changes, for example, observable differences in apoptosis rates. Apoptosis involves complex signaling pathways, and gene array data might reveal changes in upstream or regulatory genes that influence apoptosis indirectly, which may not be immediately apparent in a standard apoptosis assay. Moreover, effects on proliferation and apoptosis might not be evident under optimal cell culture conditions, which strongly foster proliferation and do not promote apoptosis. It is possible that CCL25 may only exert a significant impact under less favorable conditions, such as a pro‐inflammatory environment commonly found in OA. Further research is needed to investigate the potential influence of CCL25 under more adverse conditions.

CCL25‐stimulation induced pronounced transcriptional changes in OA MSCs, particularly within the cytokine–cytokine receptor interaction pathway. CXCR4 is the second‐highest expressed gene after stimulation with CCL25, while expression of CXCL12 in OA MSCs is downregulated by more than 10‐fold (Figure 7D). Elevated levels of CXCL12 were found in the plasma as well as synovial fluid of patients with OA [87, 88], suggesting a mechanism whereby CCL25‐mediated migration could be further enhanced by promoting the expression of CXCR4 and subsequent homing of MSCs along the CXCL12 concentration gradient. What further highlights the relevance of this finding are the results from Campbell et al. [30], who detected downregulation of CXCR4 in MSCs isolated from bone marrow lesions, which are associated with greater cartilage damage compared to isolation from lesion‐free areas. This indicates insufficient migratory capability when a certain extent of damage has been reached that could be compensated through CCL25‐mediated upregulation of CXCR4. Expression of CXCL8 and its receptor CXCR1 was also increased, potentially promoting MSCs recruitment while simultaneously raising concerns about pro‐inflammatory signaling [89, 90].

Within the TNF signaling pathway, genes such as TNFRSF1B, MMP1, and MMP14 were upregulated, supporting a role for CCL25 in enhancing migration, protecting MSCs from apoptosis, and promoting tissue repair [91–94]. However, elevated levels of PTGS1/2 suggest the potential involvement of inflammatory mediators, warranting caution [95].

Activation of the TGF‐*β* signaling pathway—as evidenced by increased expression of TGF‐*β*2, BMP2, and BMP6—may contribute to chondrogenic differentiation and cartilage regeneration [96–98]. Nevertheless, the dual role of BMPs, including their association with hypertrophic changes [99], highlights the complexity of this pathway in MSC‐mediated repair.

At the metabolic level, CCL25 activated both AMPK and PI3K/Akt/mTOR pathways, indicating metabolic reprograming to support migration. Upregulation of PFKP, EIF4EBP1, SLC7A5, and SLC3A2 reflects coordinated adjustments in glycolysis, nutrient transport, and autophagy—key mechanisms that enable MSCs to meet the energetic demands of chemotaxis and tissue regeneration [100–103]. Studies exploring the link between migration and glucose metabolism identify the CXCL12/CXCR4 axis as an essential mediator for the uptake of glucose [104], enhancement of glycolysis [105], and glucose oxidation, which eventually allows for migration toward CXCL12 [106]. It is plausible that glucose‐dependent energy generation is a prerequisite for energy‐intensive processes such as migration.

This gene array identifies genes and pathways involved in the migratory processes of OA MSCs toward CCL25 and underscores the pleiotropic actions of chemokines. A more detailed characterization of individual chemokines can help harness their regenerative functions, while also allowing for the identification of potential risks associated with their use—risks that could then be specifically addressed or mitigated.

When translated into the osteoarthritic joint environment, chemokine‐based in situ tissue engineering is expected to leverage endogenous MSC pools that remain competent and chemokine‐responsive, as shown in this study. However, OA pathology exposes transplanted constructs or targeted endogenous cells to an inflammatory, mechanically stressed, and metabolically altered microenvironment that may impair cell survival, integration, and matrix synthesis. Combining homing molecules such as CCL25 with agents that promote chondroprotection, immunomodulation, and metabolic support could improve long‐term cell viability and functional cartilage regeneration. Approaches like localized controlled release from hydrogels, costimulation of chondrogenic differentiation, or concurrent reduction of pro‐inflammatory signaling within the synovium may enhance integration into native cartilage and prolong therapeutic benefit. Ultimately, the sustained recruitment and activation of regenerative endogenous cells has the potential not only to reduce pain but also to maintain joint function and delay or prevent joint replacement in end‐stage OA.

## 5. Conclusion

Congruent to the results presented here, OA MSCs are not inferior to ND MSCs and thus constitute a suitable source to be targeted by in situ tissue engineering approaches. The chemokine CCL25 effectively recruits MSCs, and the thorough genome‐wide microarray analysis allowed for a comprehensive examination of possible involved mechanisms and pathways, posing a considerable advantage for the translation into in vivo models.

## Ethics Statement

This study was approved on the 12th of June 2014 under the title *Examination of chemokine-mediated migration of human MSCs for in situ tissue regeneration* by the ethical committee of the Charité – Universitätsmedizin Berlin (EA2/068/14) and by the patient who gave informed consent.

## Disclosure

All authors read and approved the final manuscript for publication.

## Conflicts of Interest

Michael Sittinger is an inventor of the patent: Use of chemokine‐releasing, biodegradable particles in hyaluronic acid for the treatment of cartilage defects, in particular of OA (priority DE102010062288, US9539297B2). All other authors declare that there are no conflicts of interest regarding the publication of this article.

## Author Contributions

Julia Sonnleitner, Katja Gulich, and Tilo Dehne were involved in the conception, design, data acquisition, and analysis, as well as manuscript writing. Carsten Perka, Axel Pruss, Angelika Gursche, and Daniel Kendoff contributed to data acquisition and manuscript writing. Shabnam Hemmati‐Sadeghi and Michael Sittinger were involved in conception, data interpretation, and manuscript writing. Shabnam Hemmati‐Sadeghi was involved in design and data analysis. Shabnam Hemmati‐Sadeghi and Tilo Dehne contributed equally.

## Funding

This work was supported by Bundesministerium für Bildung und Forschung (Grant 13GW0099), Else‐Kröner‐Fresenius‐Stiftung (Grant EKFS 2019T19), and Deutsche Forschungsgesellschaft (Grant DFG SI 569/7‐1).

## Supporting Information

Additional supporting information can be found online in the Supporting Information section.

## Supporting information


**Supporting Information 1** Apoptosis gating strategy: Gating strategy of OA MSCs treated with 1000 nM and stained for Annexin and PI is depicted.

**Supporting Information 2** Senescence‐associated genes: Collection of publications with genes linked to senescence.

**Supporting Information 3** Primer RT‐qPCR: List of primers used for RT‐qPCR with TaqMan and SYBR green assays.

**Supporting Information 4** Growth curve of ND and OA MSCs: The growth kinetic of ND and OA MSCs was assessed.

**Supporting Information 5** Flowcytometric measurement of surface markers of MSCs: Surface markers for the identification of MSCs were measured by flowcytometry.

**Supporting Information 6** Assessment of differentiation: Oil Red O, Von Kossa, and Alkaline phosphatase staining of ND and OA MSCs were assessed.

**Supporting Information 7** Differentially expressed genes in response to CCL25: List of differentially expressed genes in OA MSCs in response to CCL25 using an Affymetrix microarray.

**Supporting Information 8** Enriched GO terms and KEGG pathways in response to CCL25: List of enriched GO terms and KEGG pathways in OA MSCs treated with CCL25. Both lists were generated using Enrichr.

## Data Availability

Microarray data have been deposited in the National Center for Biotechnology Information Gene Expression Omnibus (GEO) and are accessible through GEO series accession numbers GSE267908 and GSE267906. Additional data are available from the corresponding author upon reasonable request.
