# Supplementary figures and images for: Osteoarthritis Bone Marrow MSCs Retain Regenerative Competence and Chemokine Responsiveness for Drug‐Based In Situ Tissue Engineering
Source: Stem Cells Int. 2025 Dec 30;2025:3757831. doi: 10.1155/sci/3757831 (PMC12767449; doi:10.1155/sci/3757831)

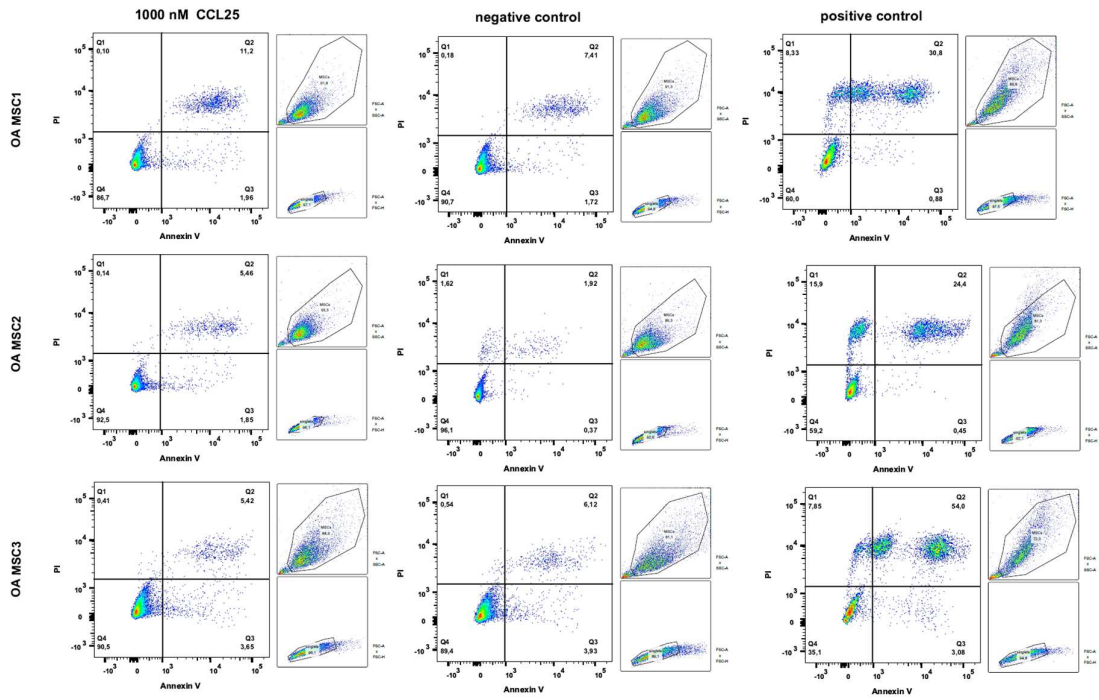

Supplement: Supplementary file 1 — Supporting Information 1 Apoptosis gating strategy: Gating strategy of OA MSCs treated with 1000 nM and stained for Annexin and PI is depicted. [file SCI-2025-3757831-s001.pdf]
