# Supplementary material for: Osteoarthritis Bone Marrow MSCs Retain Regenerative Competence and Chemokine Responsiveness for Drug‐Based In Situ Tissue Engineering
Source: Stem Cells Int. 2025 Dec 30;2025:3757831. doi: 10.1155/sci/3757831 (PMC12767449; doi:10.1155/sci/3757831)
Supplement: Supplementary file 4 — Supporting Information 4 Growth curve of ND and OA MSCs: The growth kinetic of ND and OA MSCs was assessed. [file SCI-2025-3757831-s008.pdf]

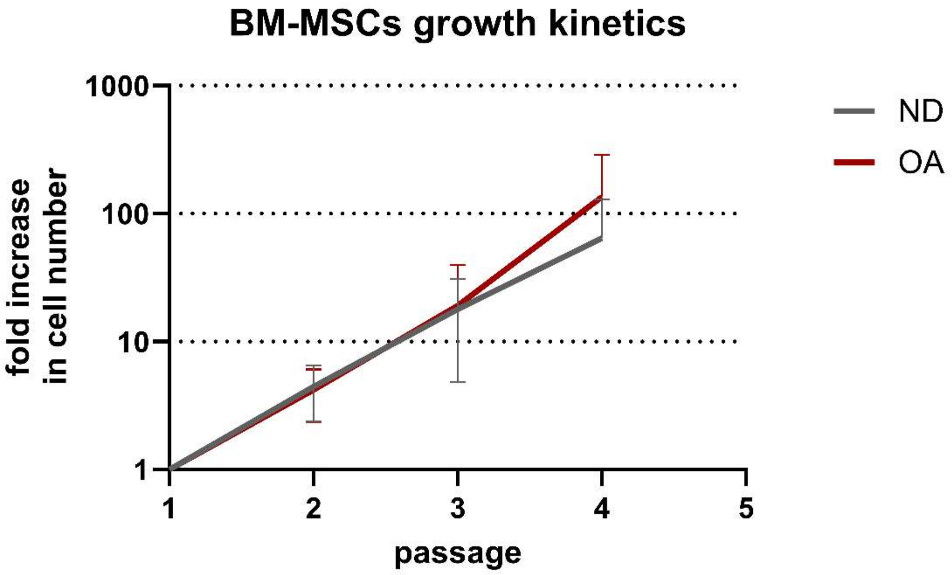

Supplementary Figure 1: Growth kinetics of BM-MSCs from ND and OA donors over 4 passages. *BM-MSCs from ND (grey line) and OA (red line) donors were expanded over four consecutive passages. Data are presented as fold increase in cell number relative to passage 1. Values represent mean ± SD of independent cultures (OA n = 25, ND n = 13).*
