# Supplementary material for: Osteoarthritis Bone Marrow MSCs Retain Regenerative Competence and Chemokine Responsiveness for Drug‐Based In Situ Tissue Engineering
Source: Stem Cells Int. 2025 Dec 30;2025:3757831. doi: 10.1155/sci/3757831 (PMC12767449; doi:10.1155/sci/3757831)
Supplement: Supplementary file 5 — Supporting Information 5 Flowcytometric measurement of surface markers of MSCs: Surface markers for the identification of MSCs were measured by flowcytometry. [file SCI-2025-3757831-s005.docx]

| **ND MSC 1** | **OA MSC 1** |
| --- | --- |
| **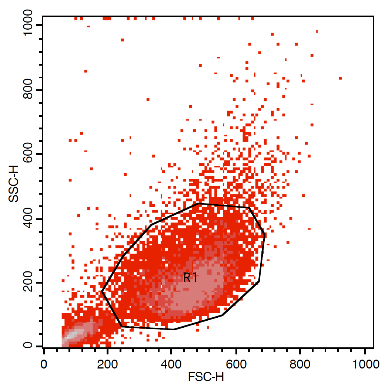** | 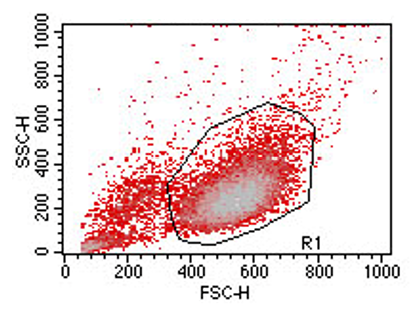 |
| **ND MSC 2** | **OA MSC 2** |
| **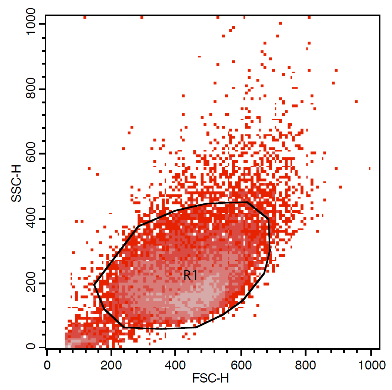** | 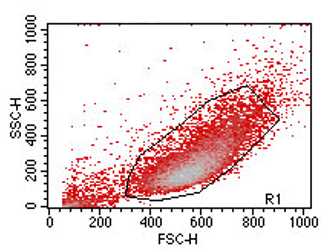 |
| **ND MSC 3** | **OA MSC 3** |
| **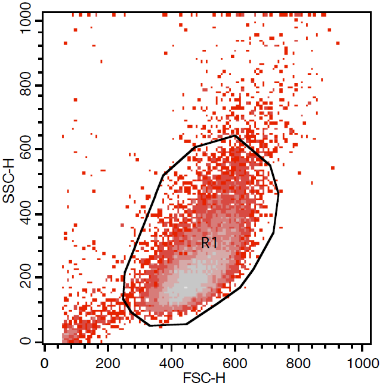** | 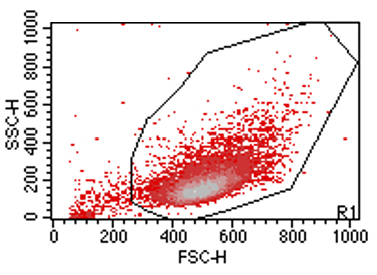 |

**Supplementary Materials -**

**Suitability of MSCs derived from osteoarthritis patients for *in situ* tissue engineering with the chemoattractant CCL25**

Figure S1: Gating strategy for the evaluation of surface marker on ND and OA MSCs.
SSC-H: side scatter – height; FSC-H: forward scatter - height

Table S1: Percentage of MSCs positive for the respective surface marker relative to the gated population

|  | OA MSC 1 | OA MSC 2 | OA MSC3 | ND MSC1 | ND MSC2 | ND MSC3 |
| --- | --- | --- | --- | --- | --- | --- |
| CD105 | 97.3% | 99.6% | 99.1% | 99.7% | 99.5% | 79.5% |
| CD90 | 94.0% | 90.5% | 99.4% | 97.4% | 99.1% | 25.8% |
| CD44 | 98.4% | 99.9% | 99.6% | 99.9% | 99.9% | 99.7% |
| CD45 | 2.5% | 0.0% | 0.1% | 0.0% | 0.0% | 0.0% |
| CD73 | 99.7% | 100.0% | 99.9% | 99.9% | 99.9% | 99.7% |
| CD166 | 76.7% | 99.8% | 99.6% | 99.8% | 99.9% | 44.1% |
| CD14 | 0.6% | 0.1% | 0.0% | 0.1% | 0.0% | 0.0% |
| CD34 | 2.5% | 0.0% | 0.0% | 0.0% | 0.1% | 0.0% |
